# Chemical Species-Dependent Structural Modification of Vitreous SiO_2_ by Monovalent Anions

**DOI:** 10.1021/acs.jpcb.5c07097

**Published:** 2026-03-19

**Authors:** Lindsay M. Harrison, Alisha N. Clark, Adam R. Sarafian, Lisa A. Moore, Craig D. Nie, Galan G. Moore, James E. Tingley, Matthew E. McKenzie

**Affiliations:** † Department of Earth Science, 1877 University of Colorado, Boulder, Boulder, Colorado 80309, United States; ‡ 2690 Corning Incorporated, Science & Technology, Corning, New York 14831, United States

## Abstract

The effects of volatile species on vitreous SiO_2_ (*v*-SiO_2_) influence transport and physical properties that have important implications for telecommunications and geological applications. While the effects of monovalent anions such as fluorine, chlorine, and hydroxyl on the transport and optical properties of *v*-SiO_2_ are highly studied, their effects on elastic properties are less well-known. We measured density and performed resonance ultrasound spectroscopy, gigahertz ultrasonic interferometry, Raman spectroscopy, Fourier transform infrared spectroscopy, and molecular dynamics simulations to obtain the elastic moduli, elastic wave speeds, structural properties, and fictive temperatures of F-, Cl-, and OH-doped *v*-SiO_2_. We find that while density reductions as a function of mol % dopant are the same for both F and Cl, the elasticity and structural data show that the addition of Cl to *v*-SiO_2_ has a buttressing effect on the network, leading to a stiffer material relative to the F-doped *v*-SiO_2_ with the formation of percolation channels. Although all three monovalent anions are incorporated into *v*-SiO_2_ by replacing an oxygen atom in SiO_4_^4–^ tetrahedra, the halogens and OH have markedly different effects on the medium-range order of *v*-SiO_2_. Poisson’s ratio and structural data show that the incorporation of halogen anions leads to closer packing of tetrahedra and an increase in the populations of smaller tetrahedral rings (3- and 4-membered) at the expense of larger tetrahedral rings (≥5-membered). In contrast, there is little distinguishable change in larger ring size in hydroxyl-doped *v*-SiO_2_ as the populations of smaller tetrahedral rings decrease, and Poisson’s ratio indicates a more open, less densely packed structure. These results imply that OH-doped *v*-SiO_2_ will be able to accommodate more stress before the onset of permanent densification than halogen-doped *v*-SiO_2_. The results of this study offer new insights into the relationship between the structure and properties of F-, Cl-, and OH-doped fused silica glasses, which are particularly applicable to the understanding of Rayleigh scattering.

## Graphical abstract

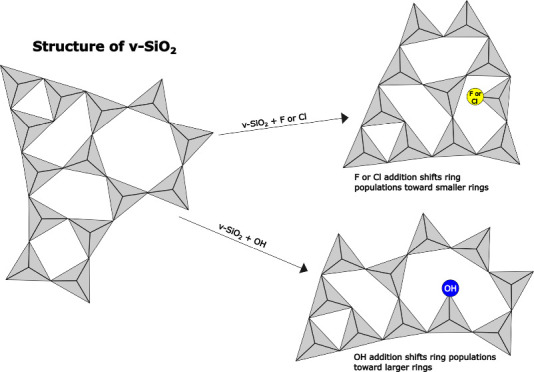

## 1 Introduction

Silica glass (*v*-SiO_2_) has many broad technical applications. Attributes of *v*-SiO_2_ can be fine-tuned to achieve technological specifications by the addition of low concentrations of dopants, or atomic species added in trace amounts. Fluorine, chlorine, and hydroxyl influence similar physical property behaviors in since they bond to similar sites (Si-X, where X is F, Cl, or OH), but these dopants are not used interchangeably in technological applications.

Chlorine and fluorine are both used in the fiber manufacturing process. For example, *v*-SiO_2_ that contains fluorine is used for optical fiber and plays an integral role in telecommunications as a refractive index modifier, reflecting light back to the fiber’s core and ensuring the signal propagates along the fiber strand. Chlorine is used as a drying agent in the manufacturing process of fused silica, where porous glass preforms are heat-treated under Cl_2_ gas atmosphere to remove OH impurities.^1^ Both fluorine and chlorine decrease viscosity, leading to lower fictive temperatures (*T*_f_) and lower density fluctuations in the glass.^2,3^ The behavior of hydroxyls in is of complementary interest. Though hydroxyl is not desired in optical fiber applications due to optical absorption in the infrared range, OH lowers viscosity and accelerates structural relaxation.^4,5^ In silicate glasses, monovalent anions act as network modifiers. They break up the silicate network by creating nonbridging bonds, ultimately lowering viscosity and allowing lower fictive temperatures to be achieved than in pure silica glass under the same cooling rate.^6,7^ However, since dopants within the same element group may have very different elastic behavior at similar concentrations, it is important to consider all these monovalent cations together.^8^

Another significant industrial use of doped *v*-SiO_2_, in bulk or in fiber, is radiation-resistant silicate glass. Whether used as shielding or as an optical fiber for in situ monitoring of spent fuel rods, it is important that glasses in these use cases are resilient to radiation. Optical fibers may provide cost-effective, real-time monitoring of below-ground storage of spent fuel rods, but their effectiveness and longevity depend on the minimization of radiation-induced attenuation, including Rayleigh scattering loss.^9^ Much research has been conducted on rare-earth and transition metal oxides on radiation-resistant glasses, but less is known on the specific effects of the contribution of the isothermal compressibility of OH and Cl.^10–12^

An important property related to the fictive temperature and elastic properties is Rayleigh scattering. Much of the research into the reduction of Rayleigh scattering loss in optical fibers has focused on reducing density fluctuations in the glass. Loss due to density fluctuations (α_ρ_) is defined as

$$\alpha_{\rho }=\frac{8\pi^{3}}{3\lambda^{4}}n^{8}p^{2}kT_{f}\beta$$ 1

where λ is wavelength, *n* is refractive index, *p* is photoelastic coefficient, *k* is the Boltzmann constant, *T*_f_ is fictive temperature, and β is isothermal compressibility, the inverse of isothermal bulk modulus *K*_T_. All three dopants in this investigation, F, Cl, and OH, are known to reduce the Rayleigh scattering. F and OH are known to cause decreases in elastic moduli.^13,14^ The addition of these network modifiers also produces measurable changes in viscosity, density, and network connectivity.^4–6,15^

In this study, we investigate how isothermal compressibility varies with monovalent anion concentration to determine whether they have measurable effects on Rayleigh scattering in *v*-SiO_2_. Here, we measured the changes in elastic properties with systematic variations in the dopant concentration. Spectroscopic data and molecular dynamics (MD) simulations are used to determine the underlying changes in the silicate network behind the elastic properties. We performed resonant ultrasound spectroscopy (RUS) and gigahertz ultrasonic interferometry on *v*-SiO_2_ containing F, Cl, and OH dopants to independently measure the elastic moduli and wave speeds, respectively. Raman spectroscopy and MD simulations of silica glasses containing fluorine, chlorine, and hydroxyl are used to interrogate structural changes in *v*-SiO_2_. Reductions in all elastic moduli across all dopants indicate that their incorporation softens the *v*-SiO_2_, but differences in Poisson’s ratio indicate different effects on the structural network. Results from Raman spectroscopy and MD simulations show changes in silicate ring structure and average Si–O–Si bond angle consistent with the observed differences in structure indicated by variations in Poisson’s ratio.

## 2 Methods

### 2.1 Sample Synthesis and Density Determination

Three suites of fused silica (*v*-SiO_2_) made by the outside vapor deposition (OVD) method containing fluorine, chlorine, and hydroxyl (OH) were prepared at Corning (Table ST1). Samples ranged from pure SiO_2_, with very low water content (<1 ppm of OH) and <10 ppb metallic impurities (Corning HPFS 8655), up to 0.42 mol % OH, 3.7 mol % Cl, and ∼5 mol % F. The fluorine- and chlorine-containing *v*-SiO_2_ was made by the laydown process, in which SiCl_4_ precursor was vaporized, then combusted using an oxygen-CH_4_ flame, where the silica soot was deposited onto a rotating rod. The resulting blank was then consolidated in a furnace and drawn into rods from which parts were cut for measurements. The hydroxyl-containing *v*-SiO_2_ glasses were made by exposing the silica soot blank to a controlled partial pressure of water in the consolidation furnace. This was achieved by passing He gas through a water bubbler held at the appropriate temperatures to achieve a range of water contents in the atmosphere. The soot blank was consolidated under a humid atmosphere to produce a glass with a known water content. One of the hydroxyl-containing *v*-SiO_2_ was made by a direct laydown flame hydrolysis (Type III) process that produces low metallic impurity, high-purity fused silica with 1000 ppm of OH (Corning HPFS 7980).

F and Cl dopant concentrations were determined using electron probe microanalysis (EPMA) (Supporting Information), while OH concentrations were measured by infrared spectroscopy. The dopants were not always distributed uniformly across the diameter of the rods; therefore, we averaged dopant concentrations across the diameter of the cane from which the RUS parts were cut, and uncertainty (standard deviation) is listed in Table S1. Density was determined on short rod (5g) samples via Archimedes’ method. The samples were then cut and rough-polished to 3 × 4 × 5 mm dimensions for RUS measurements.

### 2.2 RUS

We performed RUS on our glass samples using a Magnaflux-QRI 2600 Transceiver at Corning Incorporated. RUS obtains the entire elastic tensor from the natural resonance frequencies of the sample. Piezoelectric transducers send an ultrasonic impulse into the sample at constant amplitude but varying frequency, causing free-body resonance at the free surfaces of the sample (Figure 1). The resonance frequencies are recorded as a series of peaks through the entire frequency range of the measurement and are recorded as spectra—peak intensity vs frequency. The recorded spectra of the glass are used as boundary conditions for an inverse problem that solves for the elastic tensor. Software uses an iterative-inverse peak fitting program on a spectrum with known peaks and a known elastic tensor as estimates. The difference between the estimated and measured spectra is iteratively minimized by damped least-squares fitting. By minimizing the estimated spectra, the elastic tensor is updated to fit the measured spectra. This process is iterative until the error converges. This attribute makes RUS a very precise technique, and as such, the greatest source of error in these experiments is the uncertainty in the dimensions of the samples, which is ±2%. We used Quasar RuSpec software to perform an iterative Levenberg–Marquardt analysis method (damped least-squares) to solve the inverse problem between the calculated and measured spectra.

**Figure 1:**
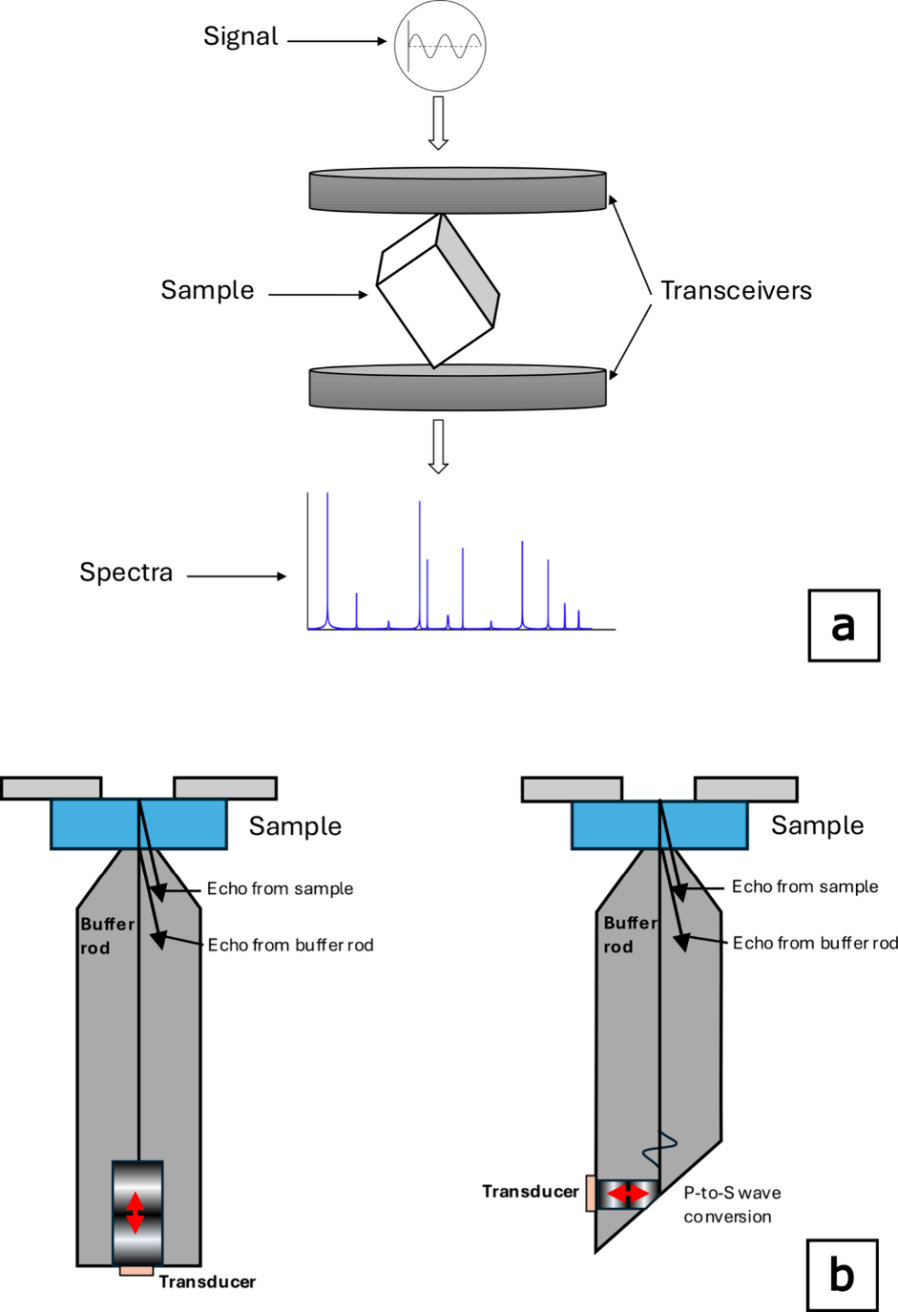
Schematics for resonance ultrasound spectroscopy (RUS) (a) and gigahertz ultrasonic interferometry (GUI) (b) methods used to determine the elastic tensor and wave speeds for doped *v*-SiO_2_ samples. (a) RUS schematic (modified from refs 18, 19 . Reference 18 is available under a CC-BY 3.0 license. Copyright 2010 Li and Gladden. Modified with permission from ref 19. Copyright 1996 Elsevier). RUS sends ultrasonic impulses across a range of frequencies that cause the free surfaces of a prism to resonate; the resonances are recorded in a spectrogram. The spectrogram is then fit using software to calculate the elastic tensor. (b) GUI schematic (modified with permission from ref 16. Copyright 2014 American Physical Society). Like RUS, this method also sends ultrasonic pulses across a range of frequencies through the sample; unlike RUS, GUI obtains the round-trip travel time of the ultrasonic pulses, which is directly related to the acoustic velocities in each sample by eqs 6 and 7.

In an isotropic solid such as *v*-SiO_2_, there are three independent values in the elastic modulus: *C*_11_, *C*_12_, and *C*_44_. The *C*_11_ and *C*_44_ tensor values correspond to the longitudinal and shear moduli, and *C*_12_ corresponds to the first Lamé parameter, λ. From these measured moduli, other elastic properties, including Young’s modulus, Poisson’s ratio, and bulk modulus, can be calculated. Young’s modulus (*E*) is calculated from the longitudinal (*C*_11_) modulus and the shear modulus (*C*_44_), *G*, as

$$E=\frac{C_{44}(3C_{11}-4C_{44})}{C_{11}-C_{44}}$$ 2

because of the equivalence in an isotropic solid of

$$C_{11}=C_{12}+2C_{44}$$ 3

This work measures the adiabatic bulk modulus (*K*_S_). At STP, *K*_T_ */K*_S_ is ∼1. This relationship is described in more detail in Section 4.3. *K*_S_ is calculated from the measured *E* and *G* (eqs 2 and 3),

$$K_{S}=C_{11}-\frac{4}{3}C_{44}$$ 4

where *C*_11_ is the longitudinal (P-wave) modulus, and *C*_44_ is *G*. Poisson’s ratio (ν) is calculated from *C*_11_ and *C*_44_ in the elastic tensor determined from RUS by

$$\nu =\frac{C_{11}-2C_{44}}{2(C_{11}-C_{44})}$$ 5

### 2.3 Gigahertz Frequency Ultrasonic Interferometry

We performed gigahertz (GHz) frequency ultrasonic interferometry at Northwestern University on F-, Cl-, and OH-containing *v*-SiO_2_ samples to measure P- and S-wave acoustic velocities. Samples were mirror-polished on parallel sides to a nominal thickness of 1 mm (thicknesses reported in Supporting Information Table S4). P- and S-wave buffer rods of single-crystal sapphire and YAG rods, respectively, are mechanically coupled directly to the samples. At the base of the buffer rods, thin-film ZnO transducers generate GHz-frequency wave packets that travel through the length of the buffer rod and into the sample.^16,17^ At each interface—buffered rod-sample and sample-free surface—a portion of the wave is transmitted, and another portion is reflected. The round-trip travel time is calculated from the interfered pulse echoes of the waves reflected at the near and far sides of the sample. Data collection is scanned in 0.2 MHz steps from 0.7 to 1.6 GHz. Round-trip travel times, traversing through twice the length of the sample, were calculated by the average frequency spacing of the maxima and minima of the interferogram. P- and S-wave velocities (*V*_P_ and *V*_S_, respectively) are calculated from the sample length (*l*) divided by the round-trip travel time (*t*) of the acoustic wave in the sample (eqs 6 and 7) and are related to the elastic moduli by the following equations:

$$V_{P}=\frac{2l}{t}=\sqrt{\frac{C_{11}}{\rho }}$$ 6

and

$$V_{S}=\frac{2l}{t}=\sqrt{\frac{C_{44}}{\rho }}$$ 7

### 2.4 Raman Spectroscopy

Raman spectroscopy was conducted at Corning Incorporated using a Horiba LabRam Evolution spectrometer equipped with an 1800 lines/mm grating and a thermoelectric-cooled CCD with 512 × 512 pixels. Spectra were obtained with a 532 nm excitation solid-state laser with a 100 mW output power, focused through a 10× objective. The acquisition time per spectrum was 20 s, and spectra were obtained from 50 to 1400 cm^–1^ at 0.53 cm^–1^ resolution. All Raman spectra were normalized to the network bending modes centered at 440 cm^–1^. We did not observe any laser-induced degradation of samples during or after data collection. Such sample degradation can be detected by drops or changes of signal intensity during accumulations and usually occurs under a higher laser output power than in our measurements.

### 2.5 Fourier Transform Infrared (FTIR) Spectroscopy

Infrared spectra of F- and Cl-doped *v*-SiO_2_ samples of 0.17 mm thickness were taken in transmission mode using a Bruker Vertex 80 brand FTIR spectrophotometer with a Hyperion 2000 microscope. The background was an open beam with a 250 × 250 um aperture. The atmosphere was purged with N_2_ gas. Spectra were recorded at 8 cm^–1^ resolution and averaged over 128 scans. FTIR data was analyzed to determine *T*_f_ from the peak position of the 2260 cm^–1^ band (υ_2_) using the equation from Agarwal and co-workers^20^ (Table S1):

$$T_{f}=\frac{43809.21}{\nu_{2}-2228.64}$$ 8

The position of the 2260 cm^–1^ band in the FTIR spectra, an overtone of the Si–O–Si stretching mode, has been shown to systematically vary with *T*_f*,*_ shifting to lower wavenumber with increasing *T*_f*.*_ ^20,21^ Studies on doped *v*-SiO_2_ have shown that this peak position is more sensitive to changes in *T*_f_ than dopant concentration, at least for F and OH concentration.^20,22^ Using the method outlined by Agarwal and co-workers, *T*_f_ can be determined using eq 8 within 15 °C.^20^ We used this peak position to determine *T*_f_ for our F and Cl samples (Figures S6 and S7 and Table S1). Density values were calculated using *T*_f_ from the relationship outlined by Gross and Tomozawa.^23^

### 2.6 MD Simulations

We performed MD simulations to study the structure and mechanical properties of F-, Cl-, and OH-doped *v*-SiO_2_ glasses at equilibrium conditions. The system was a randomly packed material consisting of 30,000 atoms. Starting from 273 K at 1 atm, the system was heated from 300 to 4000 K over 50 ps and held at 4000 K to reset the thermal history of the system. It was then cooled to 300 K to freeze the liquid structure in a glassy state. Most atomic interactions were described using the PMMCS potential, which also serves as the baseline for the pure, undoped *v*-SiO_2_.^24^ Details regarding the force field parameters, including updates to the hydrogen and generation of ab initio data for the F and Cl parameters, are provided in the Supporting Information. It is important to note that developing accurate force fields for dopants at such low concentrations typically requires extensive experimental data and training, which takes months or longer. In this study, modifications were compiled in less than 1 month for expediency, recognizing the inherent challenges in accurately modeling systems with sparse dopants. It is also important to acknowledge that the concentrations of the dopants in the simulations are different from those of the samples: lower in the F- and Cl-doped *v*-SiO_2_ and higher in the OH-doped *v*-SiO_2_.

## 3 Results and Discussion

### 3.1 Volumetric and Elastic Properties

Table S2 lists the compositions, densities, and elastic properties for all glasses in this study. These results are also plotted in Figures 2–7, and side-by-side comparisons are included in Supporting Information Figures S1–S5. Densities include both measured and calculated values, and the error was calculated from the difference between the measured and calculated density values. Fits to the elastic data as a function of concentration are calculated by a least-squares method using the NumPy function polyfit, and the slopes are given in Table 1.

**Figure 2:**
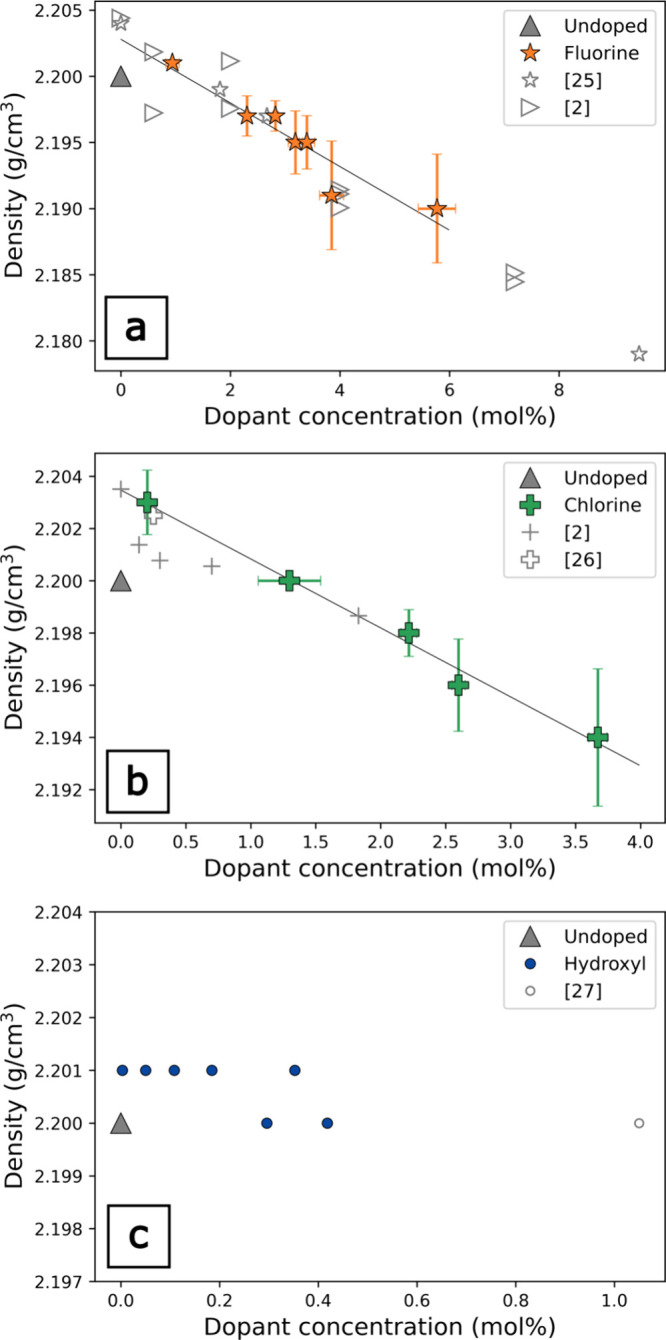
Density as a function of dopant concentration determined by the Archimedes method for F (a), Cl (b), and OH dopants (c). In (a), literature values measured by the Archimedes method^25^ and the heavy solution method^2^ for F-doped *v*-SiO_2_. In (b), literature values measured by the heavy solution method^2^ and by the Archimedes method^26^ for Cl-doped *v*-SiO_2_. In (c), literature values measured by the Archimedes method^27^ for OH-doped *v*-SiO_2_.

**Table 1:** Linear Rates of Change for Density and Elastic Properties with the Dopant Concentration

dopant	change in density (g/(cm^3^*mol))	change in Young’s modulus (GPa/mol)	change in Shear modulus (GPa/mol)	change in Poisson’s ratio (mol^–1^)	change in bulk modulus (GPa/mol)	change in *V*_P_ (m/(mol*s))	change in *V*_S_ (m/(mol*s))
F	–0.0024	–1.5	–0.67	0.001	–0.6	–49	–42
Cl	–0.0026	–1.0	–0.48	0.003	–0.2	–28	–29
OH	negligible	–0.4	–0.04	–0.005	–0.7	–55	–26

Overall, the elastic moduli decrease with increasing F, Cl, and OH in *v*-SiO_2_. The addition of volatile species (F, Cl, and OH) to *v*-SiO_2_ leads to systematic changes in volumetric and elastic properties. *v*-SiO_2_ exhibits linear reductions in density with concentration for both F and Cl of −0.0024 g/cm^3^ per mol % and −0.0026 g/cm^3^, respectively, and little change in density with OH concentration. These data are consistent with the density values of halogen-doped *v*-SiO_2_ in the literature.^2,13^ Given that the atomic weights of F and Cl are 19.00 and 35.45 g/mol, respectively, it is surprising that the density of both F- and Cl-doped *v*-SiO_2_ shows nearly the same density reduction as a function of dopant concentration, suggesting different volume expansion for the incorporation of two anions. The density of OH-doped *v*-SiO_2_ remains constant within error as a function of the concentration. Two OH-doped samples (0.3 and 0.4 mol %) have slightly lower density than the rest of the OH-doped suite. When our results are combined with the density data from Le Parc and co-workers at 1 mol % OH, a weak negative density correlation with concentration is indicated (Figure 2c).^27^ If the density trend for OH holds to a higher concentration, the density decrease of *v*-SiO_2_ due to the presence of dopants is more pronounced for the halogens than for hydroxyl.

For both F- and Cl-doped *v*-SiO_2_, there is a reduction in the elastic properties—acoustic velocities and moduli—with dopant concentration (Figures 3–6). Our results are broadly consistent with previous studies that also show reductions in the elastic moduli with F and Cl concentration.^13,26^ At relatively low dopant concentrations in the study, the error bars for the F- and Cl-doped *v*-SiO_2_ suites overlap, but there is a clear and systematic offset in the elastic moduli between the F- and Cl-doped *v*-SiO_2_. This same trend is clearly observed in the differences of the *V*_P_ and *V*_S_ for the F- and Cl-doped *v*-SiO_2_, where the velocity reductions per mol % are well outside measurement error (Figure 6). The effect of dopant concentration has the same relative effect on the elastic moduli for both F and Cl, where *E* is most sensitive to the presence of halogen dopants, exhibiting moduli reductions of −1.5 and −1.0 GPa per mol % for F- and Cl-doped *v*-SiO_2_, respectively, (Figure 3 and Table 1), and the effects of halogens on *G* are approximately half that of *E,* decreases by −0.7 GPa per mol % with the addition of F and −0.48 GPa per mol % with the addition of Cl. (Figure 4 and Table 1). *K*_S_ is the least affected modulus by dopant concentration, decreasing by −0.6 (F) and −0.2 (Cl) GPa per mol % dopant (Figure 5 and Table 1). For all elastic properties, F has a greater effect than Cl in reducing the moduli and acoustic velocities at a given concentration. Changes in *V*_P_ and *V*_S_ are shown in Figure 6a,b, respectively, and show similar relative softening between F- and Cl-doped *v*-SiO_2_, where F-doped *v*-SiO_2_ has slower wave speeds at a given dopant concentration. *V*_P_ and *V*_S_ decrease by −47 and −42 m/s per mol % with F, respectively (Figure 6 and Table 1). *V*_P_ and *V*_S_ decrease by −27 and −29 m/s per mol % with Cl, respectively. (Figure 6 and Table 1).

**Figure 3:**
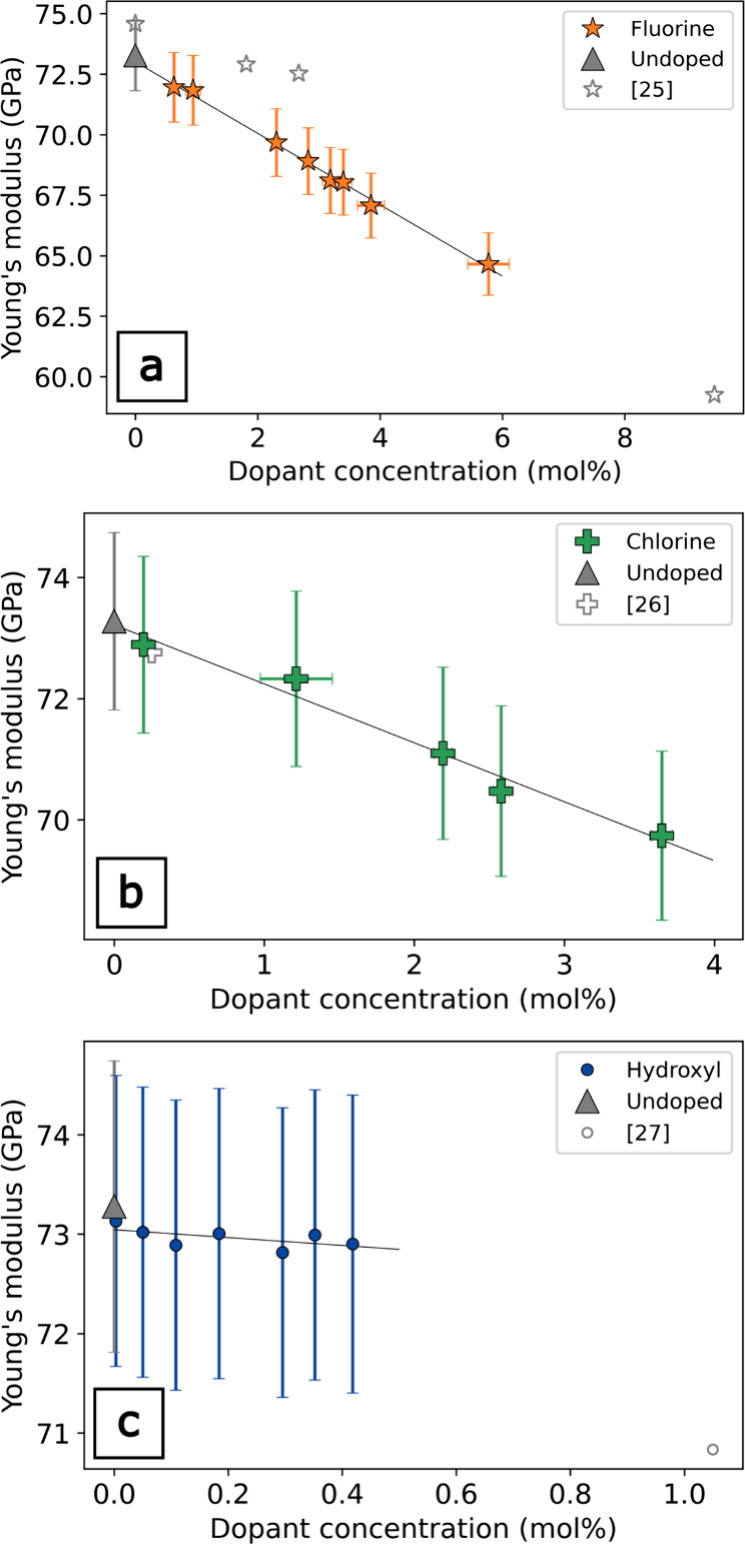
Young’s modulus as a function of dopant determined by RUS. (a) Fluorine (ref 25 determined by elastic resonance); (b) chlorine (ref 26 determined by ultrasonic microspectroscopy); (c) hydroxyl (ref 27 determined by Brillouin spectroscopy).

**Figure 4:**
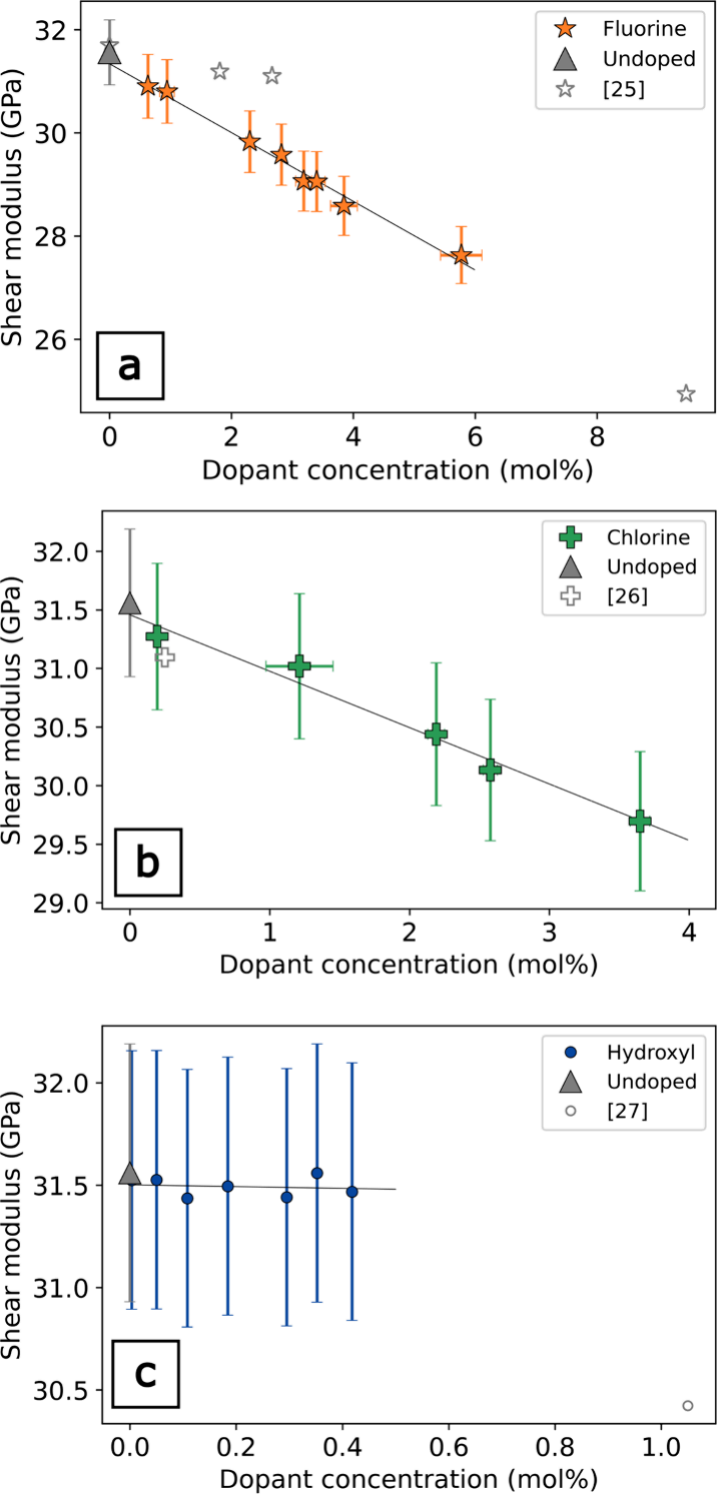
Shear modulus as a function of dopant was determined by RUS. (a) Fluorine (ref 25 determined by elastic resonance); (b) chlorine (ref 26 determined by ultrasonic microspectroscopy); (c) hydroxyl (ref 27 determined by Brillouin spectroscopy).

**Figure 5:**
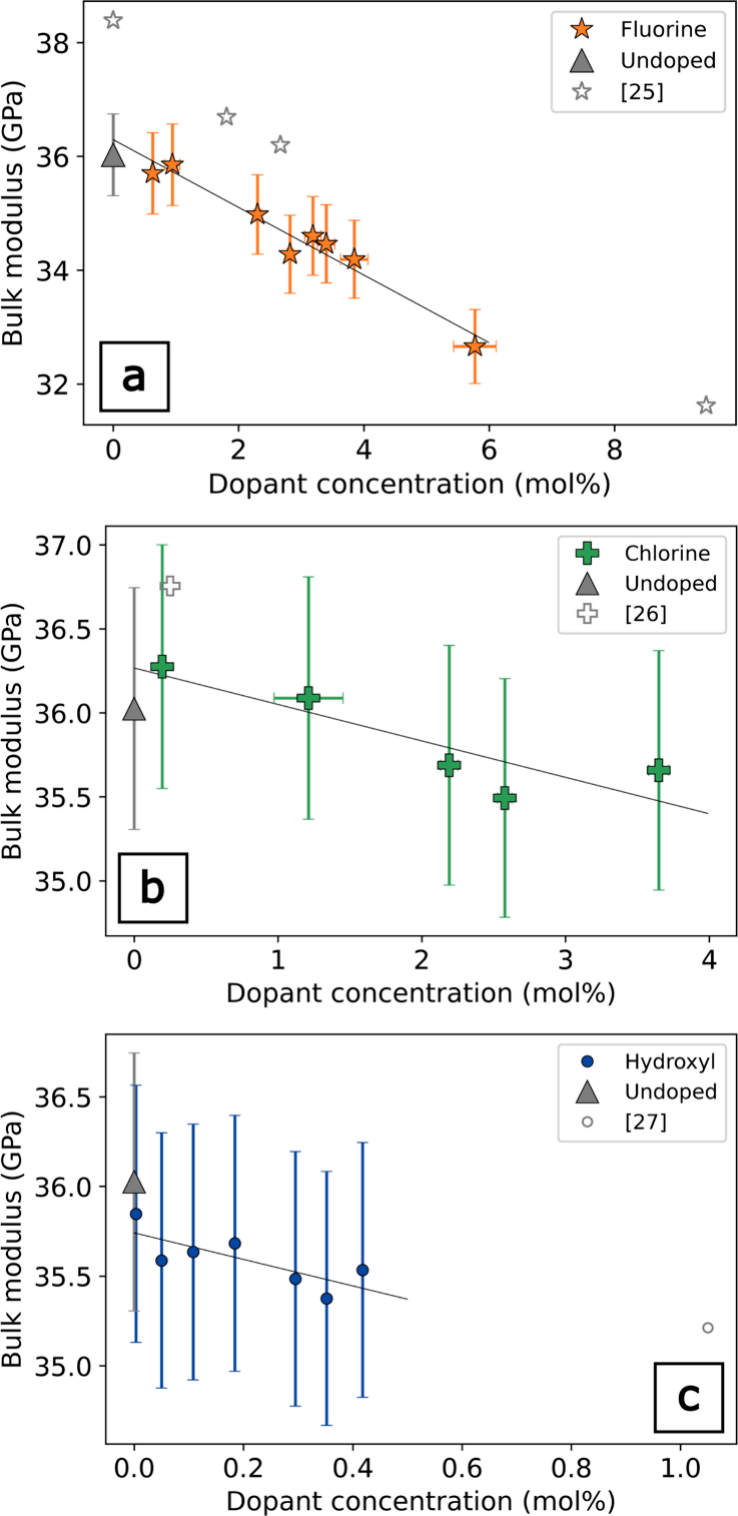
Bulk modulus as a function of the dopant determined by RUS. (a) Fluorine (ref 25 determined by elastic resonance); (b) chlorine (ref 26 determined by ultrasonic microspectroscopy); (c) hydroxyl (ref 27 determined by Brillouin spectroscopy).

**Figure 6:**
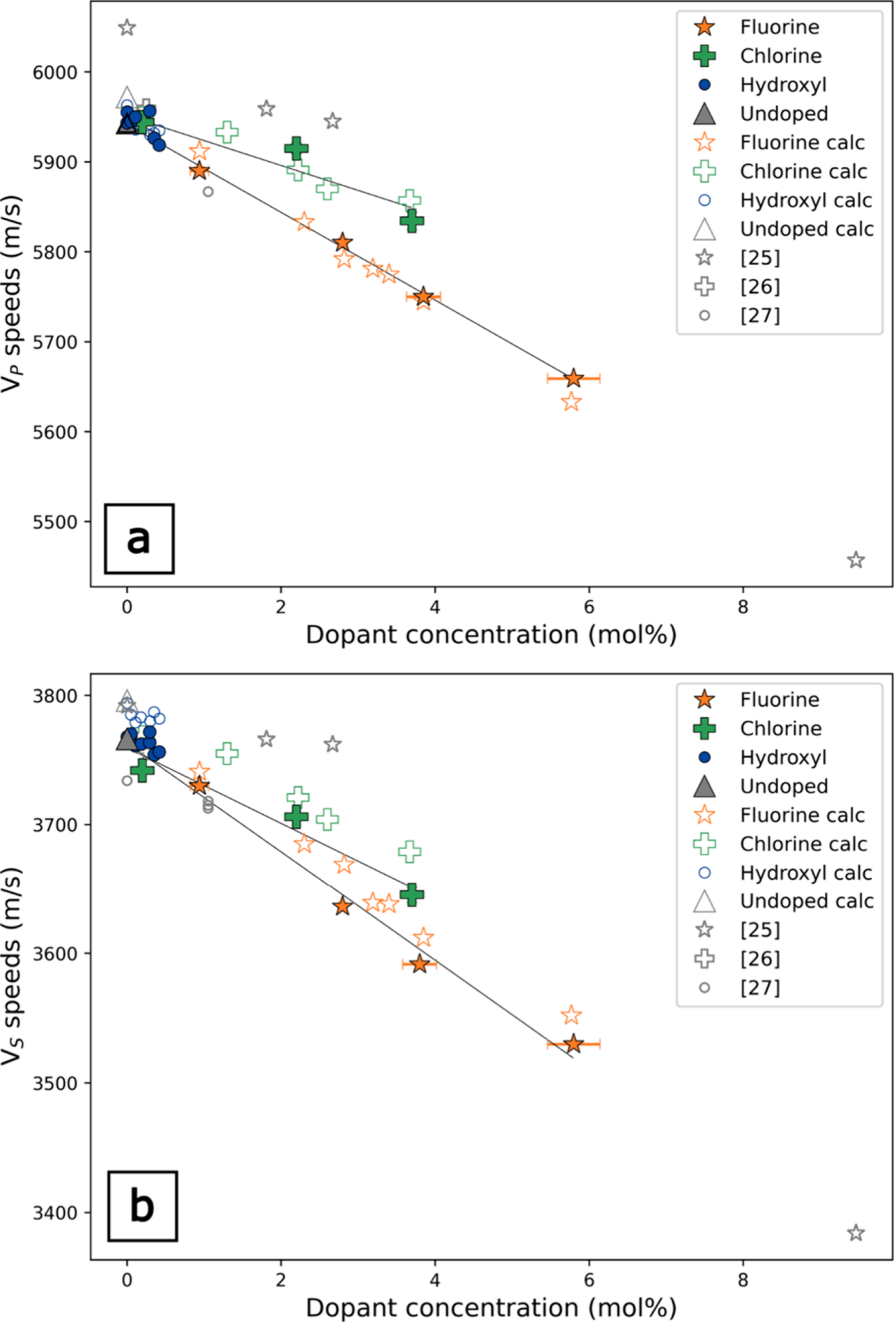
*V*_P_ (a) and *V*_S_ (b) measured by GHz ultrasonic interferometry as a function of dopant mol %, shown in filled symbols. Also shown in hollow symbols are the calculated wave speeds from RUS and density measurements given by eqs 6 and 7 in the text. Wave speed values from the literature: fluorine (ref 25 determined by elastic resonance), chlorine (ref 26 determined by ultrasonic microspectroscopy), and hydroxyl (ref 27 determined by Brillouin spectroscopy) data from the literature are also shown.

Elastic moduli and acoustic velocity are positively correlated with density—in general, denser materials are stiffer than less dense materials—and so the decrease in both density and elastic moduli with increasing dopant concentration is expected. However, a simple density dependence cannot explain the reduction in elastic properties. Density reduction per mol % is the same for F and Cl, but the elastic properties differ. This is discussed further in Section 4.1 below. The presence of an OH dopant also results in a reduction in the elastic properties relative to the undoped *v*-SiO_2_. These changes to elastic moduli with mol % OH do not follow the same trends observed for the halogens. *K*_S_ is the most reduced elastic moduli with the incorporation of OH at a given dopant concentration at −0.7 GPa per mol %, while *G* is relatively unchanged for all OH concentrations (−0.04 GPa per mol %) (Figures 4 and 5). For the OH-doped *v*-SiO_2_, the reduction in *E* as a function of dopant concentration −0.4 GPa per mol % (Figure 3). Unlike what is observed for the halogen-doped *v*-SiO_2_, *V*_P_ and *V*_S_ reductions per mol % dopant vary by a factor of 2, decreasing −54 and −25 m/s per mol % OH, respectively (Figure 6 and Table 1). It is interesting to note that *E, G,* and *V*_S_ trends for the OH-doped *v*-SiO_2_ suite indicate a stiffer material than when the halogens are incorporated, while the opposite is true for *K*_S_ and *V*_P_.

### 3.2 Poisson’s Ratio as a Function of Dopant Concentration

The effect of the dopants on the properties of *v*-SiO_2_ becomes more intriguing when Poisson’s ratio is considered as a function of dopant concentration. As shown in Figure 7, Poisson’s ratio increases with concentration for both F and Cl −0.001 per mol % F and 0.003 per mol % Cl. Linear regressions to each data set indicate that Poisson’s ratio could be increasing more rapidly as a function of concentration for Cl relative to F. However, whether this difference is physically meaningful or a result of scatter in the measurements would require a higher dopant concentration than probed in this study. It is interesting to note that the trends for concentration are more similar between F and Cl for Poisson’s ratio than for any of the individual elastic moduli or acoustic velocities. On the other hand, the OH-doped samples show a decreasing Poisson’s ratio at −0.005 per mol % (Figure 7). Our results are broadly consistent with literature values for Poisson’s ratio as a function of dopant concentration.^25–27^

**Figure 7:**
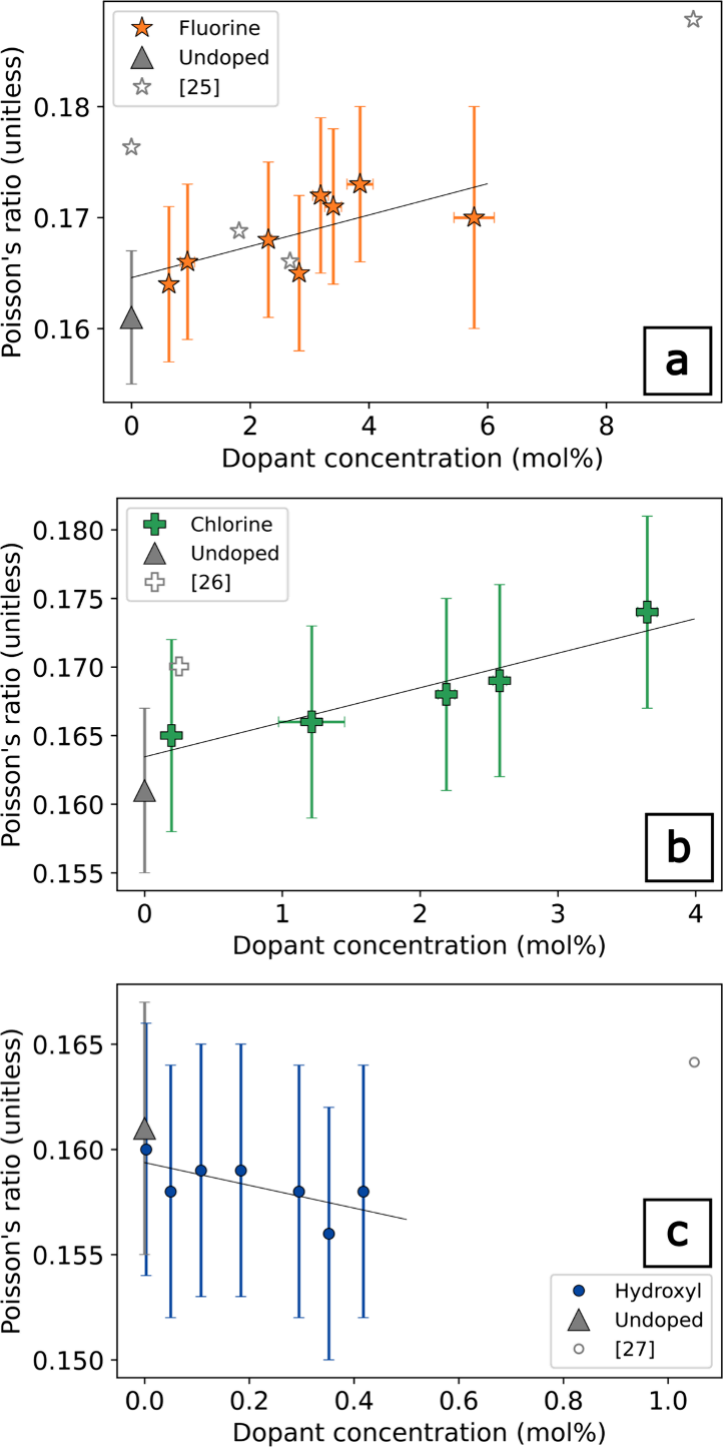
Poisson’s ratio as a function of dopant determined by RUS. (a) Fluorine (ref 25 determined by elastic resonance); (b) chlorine (ref 26 determined by ultrasonic microspectroscopy); (c) hydroxyl (ref 27 determined by Brillouin spectroscopy).

Poisson’s ratio values of isotropic materials lie between the mathematical limits of −1 and 0.5, which represent the endmembers of highly interconnected, porous structures, such as reentrant foams, and densely packed materials, such as fcc metals or rubber.^13,25–28^ At one-atmosphere, increases in Poisson’s ratio have been attributed to increased atomic packing and depolymerization of the rigid three-dimensional polyhedral network in silicate glasses.^28^ Fluorine, chlorine, and hydroxyl are all −1 charge anions; they have similar bonding behavior as they substitute into the same ionic sites in crystalline and amorphous silicates. In silicate glasses, halides and hydroxyl bond to Si, creating Si–X groups (where X = F, Cl, and OH), disrupting the polymerized network of SiO_4_ tetrahedra and creating a nonbridging oxygen (NBO).^7,29–31^ Increasing Poisson’s ratio values for F- and Cl-doped *v*-SiO_2_ indicate that the structure is becoming increasingly close-packed, in contrast to the OH-doped *v*-SiO_2_. As all dopants bond to the same site, the difference in Poisson’s ratio is not due to differences in degrees of depolymerization at a given concentration. Rather, this suggests that there are significant differences in the modifications to the silicate network by the incorporation of halogens and hydroxyl.

Decreases in Poisson’s ratio exhibited by the OH-doped *v*-SiO_2_ indicate that the structure of *v*-SiO_2_ becomes more open and interconnected with increasing concentration of OH. Additionally, the large effect on *K*_S_ with OH concentration stands in contrast to the halogen dopants, where *K*_S_ is the least reduced moduli with dopant concentration (Table 1 and Figure 4a). These indicate that stress in OH-doped *v*-SiO_2_ is accommodated by network flexibility of the rigid SiO_4_ tetrahedra.^16,32^ The lack of long-range order, high degree of polymerization, and open network leads to volume reduction that is accommodated by the bending and buckling of rigid polyhedra relative to one another. This network-style structure may explain the lack of change in *G* observed and the lower effect on *V*_S_ velocities relative to *V*_P_ with increasing OH concentration because silicate networks are less prone to shearing deformation as they become more open.^33^

### 3.3 Spectroscopic Results and Structural Properties

Raman spectra for all doped *v*-SiO_2_ glasses are shown in Figure 8, with the pure, undoped *v-*SiO_2_ spectrum shown in the gray dashed curves in Figure 8 and also with major features identified in Figure 9a. Difference spectra in Figure 9b–d were calculated by subtracting the undoped silica spectrum from each individual doped silica spectrum. There are four Raman peaks that exhibit significant modification with concentration across all samples: the boson peak, the R-band, and the D1 and D2 peaks (Figure 8). The boson peak located at 60 to 120 cm^–1^ is correlated with long-range structural disorder.^34^ The R-band centered at ∼440 cm^–1^ corresponds to the ≥5-member ring population.^35,36^ The D1 and D2 peaks at 495 and 606 cm^–1^ are due to the presence of 3- and 4-membered silica rings, respectively.^37,38^ Figure 9 shows the difference spectra of all doped *v*-SiO_2_, with color intensity increasing with dopant concentration.

**Figure 8:**
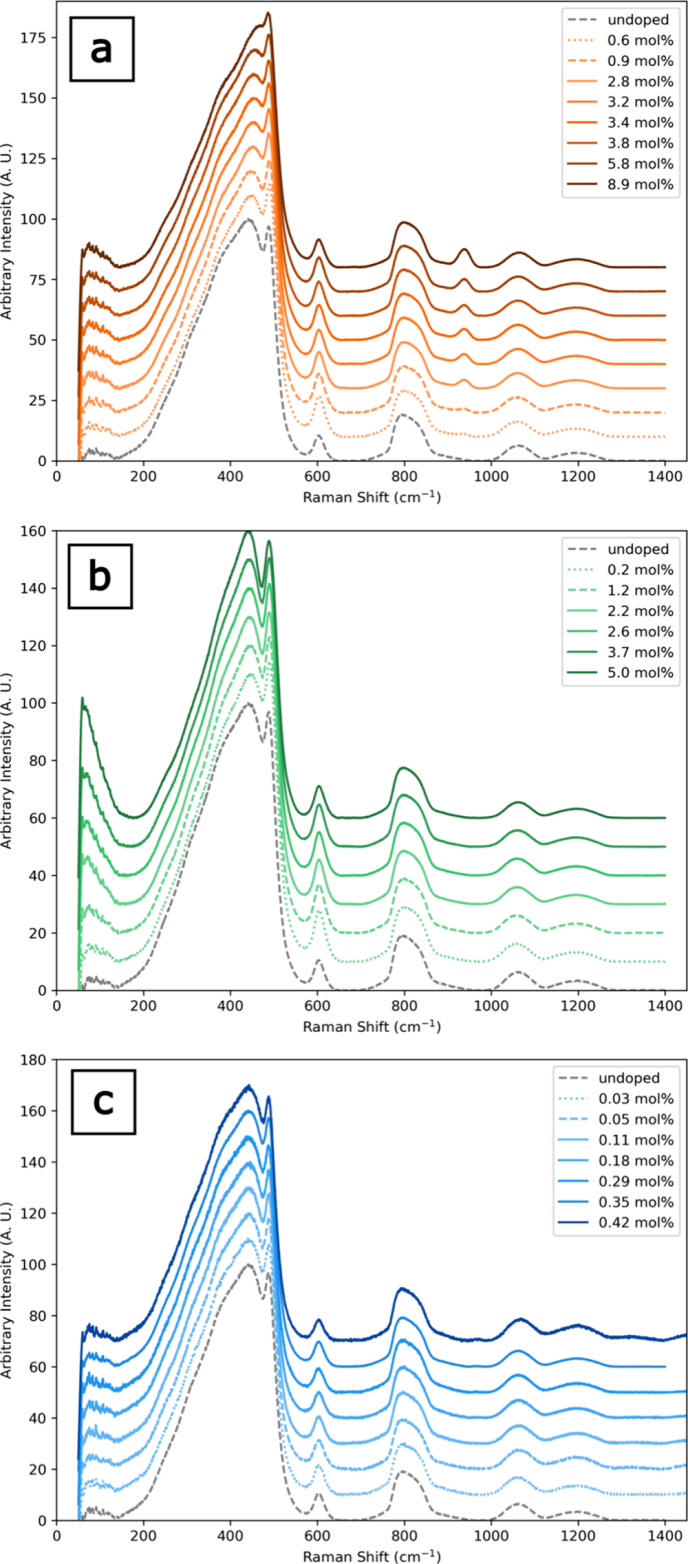
Full Raman spectra of F (a), Cl (b), and OH-doped (c) *v*-SiO_2_ samples. Spectra are offset to emphasize changes in the spectrum across samples of each dopant suite.

**Figure 9:**
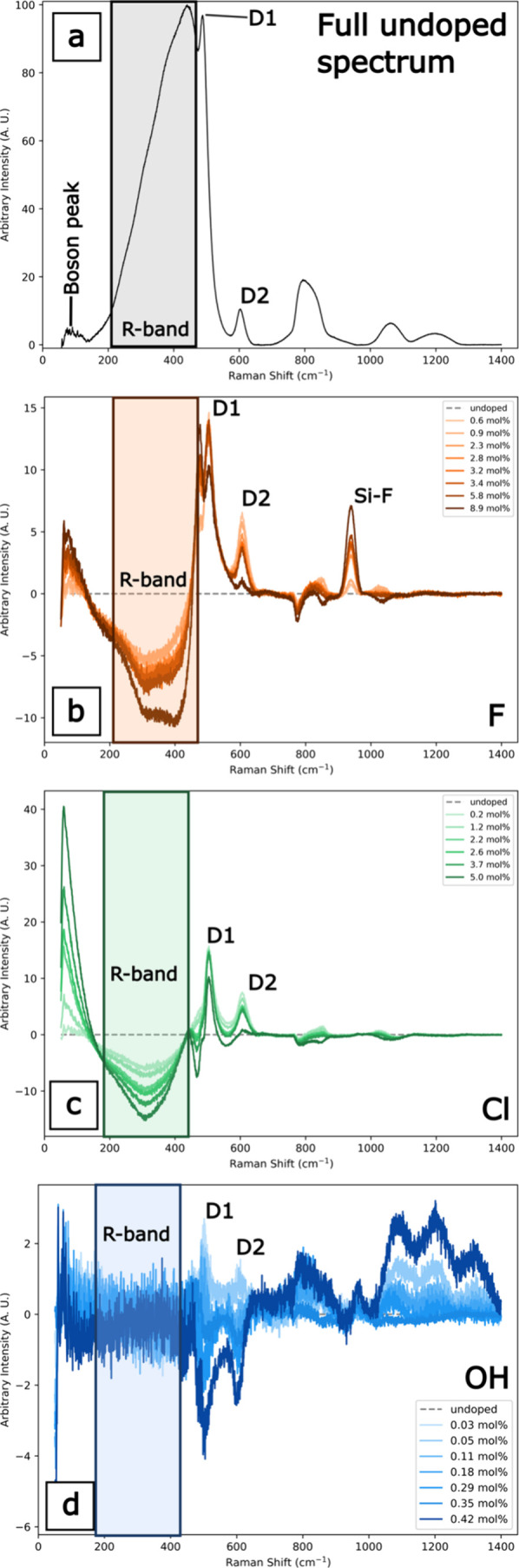
(a) Raman spectra of pure undoped *v*-SiO_2_. Difference Raman spectra of F (b), Cl (c), and OH (d) doped. The difference is taken from each doped silica spectrum and the pure silica spectrum so that the pure silica difference spectrum maintains a constant baseline at 0 intensity. Both the F- and Cl-doped silicas have nearly the same amplitudes for the D1 peak.

Difference spectra illuminate the structural modification with increasing dopant concentration at a given wavenumber and are calculated by subtracting the undoped silica spectrum from each individual doped silica spectrum (Figure 9). The boson peak intensity increases with increasing mol % F and Cl, but not OH; the effect is especially pronounced in the Cl-doped *v*-SiO_2_ (Figure 9c). There are decreases in the R-band intensities in the F- and Cl-containing *v*-SiO_2_ spectra, but no observable change in intensity in the OH-containing *v*-SiO_2_ spectrum (Figure 9d). In the halogen-containing *v*-SiO_2_ spectra, we observe increases in the intensities of the D1 and D2 defect bands relative to the undoped *v*-SiO_2_. Increases in these band intensities correspond to increases in 3- and 4-member ring populations. The intensities of these bands, which are governed by *T*_f_ and not dopant concentration,^21^ follow the trends in *T*_f_ as outlined in Table S1, where the undoped silica has a *T*_f_ much lower than the highest F concentration. The D1 and D2 band intensities only decrease with increasing OH concentration and are lower than the undoped *v*-SiO_2_, indicating a decrease in the 3- and 4-member silica rings with increasing mol % OH.

Raman spectra of the F-doped samples also show a symmetric peak at 945 cm^–1^ whose intensity increases with F concentration (Figure 9b). This band has been correlated with the Si–F stretching mode.^39^ This peak shows that F bonds directly to Si, mostly forming SiO_3/2_F (and some SiO_4/2_F) tetrahedra at the expense of a SiO_4_ tetrahedra, which has been observed in NMR data.^7^ Raman spectroscopy measurements are useful to determine whether the structural modification of the silicate network, with the incorporation of F, Cl, and OH, is consistent with the differences observed in Poisson’s ratio. In the low frequency region (200–700 cm^–1^), the R-band is related to the presence of ≥5-member SiO_4_ tetrahedra rings, while the D1 and D2 defect bands correspond to the breathing modes of 4- and 3- member rings, respectively.^35,36,38,40^ Raman spectra, both full and difference, show decreases in the R-band intensity and increases in the D1 and D2 intensities for all concentrations of F or Cl relative to the undoped *v*-SiO_2_ (Figure 9). This indicates that the addition of halogens results in fewer large rings. In contrast, the Raman data for the OH-doped *v*-SiO_2_ show no discernible change in the R-band and decreases in the D1 and D2 bands relative to the undoped *v*-SiO_2_ (Figure 9), also indicating decreases in the populations of small 3- and 4-member rings.

### 3.4 Simulation Results

The determination of the ring size distribution and the Si–O–Si (intertetrahedral) bond angle as a function of dopant compositions was determined by MD simulations and is shown in Figure 10. For comparison, undoped *v*-SiO_2_ exhibits narrower ring size and intertetrahedral angle distributions relative to the doped *v*-SiO_2_, which is consistent with a high Poisson’s ratio value compared to experimental values for the undoped sample (Table S2). Like our Raman spectroscopy results, our MD simulation results show that, with increasing F and Cl concentration, the population of small 3- and 4-member rings increases (Figure 10a,c). The intertetrahedral angle distribution in F- and Cl-doped *v*-SiO_2_ broadens with the concentration, as shown in Figure 10b,d. For F-doped *v*-SiO_2_, the largest populations of intertetrahedral angles appear around 125–150°, but the peak decreases in population as F concentration increases, and the population of smaller (between 75 and 125°) angles increases with concentration. The broadening of this distribution indicates that the average intertetrahedral angle is decreasing with increasing F concentration. A reduction in average ring size corresponds to a tightening of the intertetrahedral angle. Supporting this interpretation, the MD simulations show that as the concentration of Cl increases, the distribution of ring sizes shifts from higher populations of larger rings (>7-member) rings to smaller (3- to 6-member) rings, with the population of 7-member rings remaining constant (Figure 10c). There is a slight shift in the Si–O–Si angle from a symmetric to an asymmetric Gaussian distribution with increasing Cl concentration, with a decrease in larger angles and a slight increase in smaller angles (Figure 10d). This is consistent with an increase in the number of small member rings as the intertetrahedral angle is lower in smaller rings. At high concentrations of OH, MD simulations shift to larger rings with the addition of OH (Figure 10e); ring sizes decrease at smaller (<5-member) rings and increase at larger (≥5-member) rings. These simulations show increases in ring size that were not observed in the Raman data, perhaps because the OH concentrations in the samples are not high enough to observe changes in the R-band from the noise. There is no significant change in the distribution of Si–O–Si angles with increasing OH concentration (Figure 10f), consistent with a decrease in the number of 3- and 4-membered ring populations.

**Figure 10:**
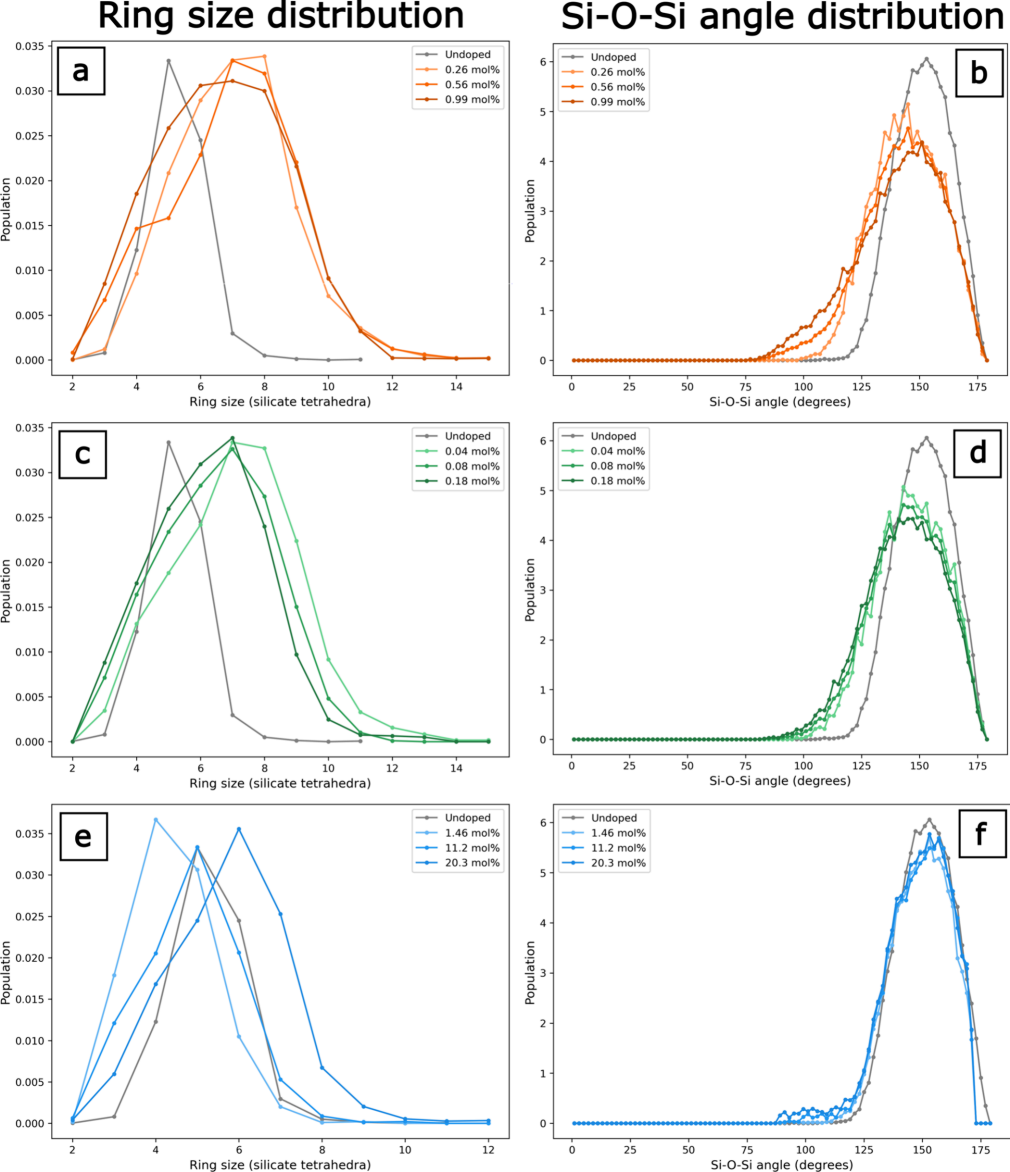
Ring size distribution (left) and Si–O–Si angle distribution (right) determined from MD simulations of fluorine- (a, b), chlorine- (c, d), and hydroxyl-doped (e, f) silicas.

Importantly, the results of our MD simulations provide information on the changing populations of 3- and 4-membered rings as a function of dopant concentration, not fictive temperature; in this regard, the MD simulations can isolate the 3- and 4-membered ring population changes than the D1 and D2 peaks in Raman spectra for glasses with the same *T*_f_. These results are consistent with our elastic and spectroscopic measurements, reinforcing the findings that the incorporation of F and Cl in *v*-SiO_2_ leads to larger populations of 3- and 4-member rings that lead to increased network flexibility and a more tightly packed and relatively more uniform structure. Conversely, the MD results show that the incorporation of OH in *v*-SiO_2_ leads to larger populations of ≥5-member rings, which also lead to increased network flexibility but yield a more open structure.

### 3.5 Evaluating the Effect of Fictive Temperature on Volumetric and Elastic Properties

Fictive temperature (*T*_f_) is the temperature at which the structure of the supercooled liquid is frozen into the glass on cooling. The structure of the glass is the same as that of the liquid at *T*_f_. *T*_f_ is around 1200 °C for pure, dry *v*-SiO_2_. However, *T*_f_ can vary widely, depending on the cooling rate or annealing. The density of *v*-SiO_2_ anomalously increases with *T*_f_ from 950 to 1500 °C.^41^ The addition of dopants like F, Cl, and OH is known to enhance structural relaxation and lower *T*_f_ for doped *v*-SiO_2_.^4,42,43^ As lower *T*_f_ corresponds to lower densities, the following question arises: could the trends we observe for *v*-SiO_2_ just be an effect of *T*_f_?

For all sample suites, the 2260 cm^–1^ peak shifts to a higher wavenumber with increasing dopant concentration, indicating that *T*_f_ decreases with increasing dopant concentration. As stated above, this is to be expected, as the presence of dopants enhances structural relaxation in *v*-SiO_2_.^4,42,43^ If changes in *T*_f_ were controlling the density reductions, then given that there are differences in *T*_f_ between the halogen-doped *v*-SiO_2_ samples, it would be expected that the density reduction as a function of mol % would be greater for F than for Cl, which is not observed. Using the density change as a function of *T*_f_ equation from ref 23 for *v*-SiO_2_, the Cl-suite would have effectively flat density values (Figure S1) due to the small range in *T*_f_, which is not observed (Figure 2). Also, the density trend for F should be nonlinear as a function of concentration, given the reduction in *T*_f_ with increasing F concentration. Furthermore, if the *T*_f_ is below 950 °C, then there should be an inflection point with density increasing at low *T*_f_, given the density minimum at 950 °C^41^ and inflection for density as a function of *T*_f_ at 3.3 mol %,^22^ which is not observed in our data. Also, the anomalous densification of *v*-SiO_2_ has been demonstrated to arise from the coexistence of two polyamorphic states that are comprised of predominantly 6-member rings, with the main structural difference being the variation in intertetrahedral angle.^16,33,44^ Our Raman and MD data show that for all samples, there is significant modification to the average ring size, and moreover, the structural changes are dependent on the atomic species of the dopant, with the presence of halogens increasing the populations of <6-member rings, while the presence of OH decreases these populations. This is not to say that *T*_f_ plays no role in the density, structural, or elastic properties of F-, Cl-, and OH-doped *v*-SiO_2_, but rather that structural relaxation (and thus lower *T*_f_) does not appear to be the dominant control on the similarities of the halogen-doped *v*-SiO_2_ or the difference between the halogen- and OH-doped *v*-SiO_2_.

## 4 Implications and Applications

### 4.1 Chlorine Buttresses the Silicate Network

The decoupling of volumetric and elastic properties is apparent from the comparison of the density and the elastic moduli for the doped *v*-SiO_2_ (Figures 2–5). As mentioned above, all dopants are bonded to the same atomic site in the *v*-SiO_2_ structure. Density reductions as a function of dopant concentration are independent of the chemical species. Poisson’s ratios with increasing dopant concentration are well within error, suggesting that the degree of network connectivity is comparable at the same concentration. However, there are clear differences in elastic moduli and acoustic velocities across dopant species. At a given dopant concentration, F always has a more pronounced effect on lowering the elastic moduli and the acoustic velocities compared to Cl. If the reduction of elastic moduli and acoustic velocities was due solely to the depolymerization of the network of SiO_4_ tetrahedra by the incorporation of −1 anions, it would follow that the effect of incorporation of F and Cl would be the same since the density reductions are the same as a function of concentration. However, this is not observed (Figures 3–5).

As mentioned above, the reductions in elastic moduli and acoustic velocity are ∼1.5× greater for F relative to Cl. This relative relationship between F- and Cl-doped *v*-SiO_2_ is the opposite of what is observed in crystalline salts, where the F compound always has a stiffer response relative to the Cl compound.^45^ In crystalline materials, the incorporation of the larger anion expands the unit cell, resulting in longer atomic bonds and a more compressible material, even though the density is higher for the Cl-salts relative to the F-salts. The observed difference in elastic response between the halogens in *v*-SiO_2_ may be due to the difference in atomic radii but not because of larger average ring size, bond angle, or bond lengths. The atomic radius of the Cl^1–^ anion is 1.8 Å, approximately 1.5× larger than the 1.2 Å F^–1^ anion. It is intriguing that the radii difference between the anions is the same as the difference for *E*, *G*, and acoustic velocity. The larger Cl anion may buttress the open ring structure of *v*-SiO_2_, stiffening the silicate network and increasing the apparent atomic packing. If so, the buttressing of the network of rigid polyhedra could account for the 3× increase in *K*_S_ and Poisson’s ratio for the Cl-doped *v*-SiO_2_ relative to the F-doped *v*-SiO_2_. Noble gases have been shown to occupy the interstitial void space in silicate glasses at high pressure, leading to a stiffer response on compression.^46,47^ Perhaps a similar effect is occurring with the incorporation of Cl in the structure of *v*-SiO_2_, where the large Cl anion adds some internal physical support to the interconnected network SiO_4_, resulting in higher moduli for the Cl-doped *v*-SiO_2_ relative to the F-doped *v*-SiO_2_.

The boson peak has been correlated with structural disorder and density fluctuations.^34,48^ As an example, Raman spectroscopy of alkali-doped aluminosilicate glasses shows increasing intensity of the boson peak as K replaced Na in the glass and is attributed to increasing the size of tetrahedral rings and cages as the larger K atom replaces the smaller Na atom.^49^ In our spectra, the boson peak intensity has a positive correlation with F content, as it does with Cl content. The presence of Cl has a greater effect on the intensity of the boson peak as a function of concentration (Figure S8). At 5 mol % F, the silica in our study containing the most F, the change in boson peak intensity from the undoped silica tops out at about 5 a.u., whereas for 2.6 mol % Cl, the boson peak intensity change from undoped reaches 40 a.u., which is 8 times higher than that of the F intensity. However, we see similar shifts to a smaller ring size population from Poisson’s ratio, the R-, D1, and D2 bands in the Raman spectra, and from the ring-size determination from the MD simulations. Despite Cl having similar bonding and macroscopic/physicochemical effects on the silicate network as F, the increased intensity of the boson peak suggests that percolation channels—clusters or column-like regions within the silicate network forming extended tunnels—are more readily formed in Cl-doped *v*-SiO_2_. This may result from the larger size of the Cl^–^ anion, which distorts and expands the network cages to a greater extent than does F^–^, thereby promoting the development of interconnected pathways that are less prevalent or absent in F-doped *v*-SiO_2_.

### 4.2 Implications for SiO_2_ at High Pressures and Temperatures

The main structural feature of amorphous silicates is the three-dimensional interconnected network consisting of rigid silicate tetrahedra. While we observe decreases in *E* and *K*_S_ and varied-to-no decreases in *G* for all dopants, it is the Poisson’s ratio that is the most indicative of structural changes in the glass. As mentioned previously, high Poisson’s ratio values (approaching 0.5) correspond to densely packed structures, such as metals or rubber; low Poisson’s ratios (approaching −1) are typical of highly porous, reentrant materials, such as cork, foams, and aerogels.^28^ Poisson’s ratio of halogen-doped *v*-SiO_2_ in this study slightly increased with concentration (Figure 7), indicative of increasingly densely packed structures. Interestingly, the increase in Poisson’s ratios for the halogen-doped *v*-SiO_2_ is not accompanied by increases in density. Marians and Hobbs determined that smaller rings of SiO_4_ tetrahedra are associated with lower densities, whereas increasingly larger rings are associated with increased density.^50^ While increases in density are typically associated with decreasing interatomic spacing, increasing Poisson’s ratio values are not necessarily indicative of more closely packed atoms but may be explained by increasing populations of smaller rings and cages.

A Si-anion group and a nonbridging oxygen (NBO) pair form when halides and hydroxyl are incorporated in the amorphous silicate network.^7,30,31,47^ There may be distinct behavior differentiating the halogens from hydroxyl, such as in our Poisson’s ratio data, but the trend is within error and may warrant further investigation (Figure 7). Decreases in elastic modulus with increasing F content at low temperatures have been correlated with decreased polymerization observed by ref 13. Tightening of intertetrahedral angles and increased numbers of smaller rings, which correspond to closer packing of a material, are both supported by our observations of increasing Poisson’s ratio indicative of a closer-packing material.^28^ This behavior is also reflected in MD simulations of the intertetrahedral angle and ring size (Figure 10). As the data for F- and Cl-doped *v*-SiO_2_ indicate increases in the populations of smaller rings as well as tightening of intertetrahedral angles with increasing halogen content, stress on the silicate network may be accommodated by the smaller angles, as they have been shown to accommodate deformation in high pressure studies.^51,52^ In contrast to the halogen-doped *v*-SiO_2_, it is clear that the modulus changes for hydroxyl-doped *v*-SiO_2_ are caused by an entirely different structural mechanism.

Typically, glasses become more compressible (i.e., lower bulk modulus) with more openness in the silicate network, as is observed in the hydroxyl-doped *v*-SiO_2_, which takes on a more open, less dense structure with OH incorporation.^27^ As more hydroxyl is introduced to silica, the NBO formation leads to higher populations of larger, more open rings as incorporation of OH hydrolyzes the structure and interrupts bonds, creating NBOs. Network stresses are accommodated by bending of intertetrahedral angles and rotation of Si–O–Si about the Si atoms.^44^ As intertetrahedral angles approach a minimum angle with increasing pressure,^53^ larger rings are found to be more stable formations at high pressure, as they are able to accommodate more densification.^33,54^ While the interstitial voids themselves may be the reason for larger changes in *C*_11_ and *K*_S_ as they both depend on density variations propagating through the material (compressional body waves), the relative rigidity of the wider intertetrahedral angles and larger rings may be responsible for the smaller changes in *C*_44_ (*G*) relative to halogen-doped *v*-SiO_2_.

Both halogen-doped and hydroxyl-doped *v*-SiO_2_ have different mechanisms to accommodate stress in the silicate network that increases as dopant concentration increases up through the concentrations we measured. Due to the nature of the stress accommodation mechanism, hydroxyl-doped *v*-SiO_2_ can accommodate much larger stresses before the network will have to break and reform bonds to maintain stability under pressure. This means that we would predict that the onset of permanent densification in *v*-SiO_2_ should occur at pressures lower than those observed in undoped or OH-doped *v*-SiO_2_.

### 4.3 Application to Rayleigh Scattering

Rayleigh scattering is an important source of optical loss in optical fibers and optical elements for ultraviolet applications, such as deep UV lithography lenses. In designing optical fibers or other *v*-SiO_2_-based materials, one must carefully balance the dopants to achieve the desired optical performance (e.g., low signal loss) while maintaining enough mechanical strength to withstand stress. In optical fibers, fluorine is used as a refractive index suppressor in the cladding of pure silica core fibers. Cl may be used as an index raiser or may be present as a residual species. OH present in fused silica used for UV applications affects both transmission and laser damage behavior.^55^ Therefore, it is important to understand how these dopants affect Rayleigh scattering in order to minimize losses.

Equation 1 describes the Rayleigh scattering loss due to density fluctuations in the glass. Recall that β is isothermal compressibility, the inverse of *K*_T_, the isothermal bulk modulus. The conversion of adiabatic bulk modulus, *K*_S_, which we obtained from RUS to *K*_T*,*_ is given by the following equation:

$$K_{T}=K_{S}(1+\alpha \gamma T)$$ 9,

where *T* is temperature, γ is the Gruneisen parameter, given by

$$\gamma =\frac{\alpha K_{S}}{\rho C_{P}}$$ 10,

where α is the thermal expansion coefficient, ρ is the density, and *C*_P_ is the specific heat at constant pressure. For undoped fused silica, α is 5 × 10^7^, *K*_S_ is 36.0 GPa, ρ is 2.20 g/cm^3^, and *C*_P_ is 0.770 J/(g K) (Corning fact sheet). For *T* = 300 K, eq 9 gives *K*_T_ ≈ 36 GPa. Because *K*_T_ ≈ *K*_S_, the inverse of our bulk moduli, can be directly applicable to the Rayleigh scattering equation.

The trends in *K*_S_ for F-, Cl-, and OH-doped *v*-SiO_2_ shown in Table 1 are the inverse of the compressibility trends. OH-dopant increases compressibility the most; the F-dopant has a similar, although lower, effect on increasing compressibility in *v*-SiO_2_. Cl-doped *v*-SiO_2_ is the least compressible by a factor of ∼3 less than OH and F. Scattering loss due to density fluctuations is linearly correlated to both compressibility and *T*_f*,*_ but our results show that *T*_f_ does not alone explain changes in elastic and structural properties in F-, Cl-, or OH-doped *v*-SiO_2_. Changes in compressibility at these concentrations will increase signal loss due to Rayleigh scattering by density fluctuations by up to ∼10% calculated from the difference between the inverse of the isothermal bulk modulus between the 5 mol % F *v*-SiO_2_ sample and that of the undoped *v*-SiO_2_ sample. On the other hand, the increase in Rayleigh scattering due to density fluctuations for the 3.67 mol % Cl sample is less than 1% and well within measurement error. Though the range of compositions for OH-doped *v*-SiO_2_ in this study is far lower than that of Cl, the increase in Rayleigh scattering is 1.4%, which is greater than that of Cl but also still within error. Of all dopants, Cl produces the smallest reductions in all elastic moduli, indicating that it not only has lower optical signal loss due to isothermal compressibility than F and OH, but may also be more resistant to mechanical stresses.

## 5 Conclusions

Taken together, the volumetric and elastic moduli data suggest that while F, Cl, and OH all bond to Si, there are significant differences in the way the bulk structure of *v*-SiO_2_ incorporates these monovalent anions. We leverage experimental data and MD simulations to support a new conceptual framework that outlines how different monovalent anion species affect the medium-range order in *v*-SiO_2_. Our key findings clarify how each dopant uniquely affects the network structure and mechanical properties of *v*-SiO_2_.

1. Density reductions per mol % for Cl and F are effectively indistinguishable.
2. The measured elastic moduli and wave speeds of F- and Cl-doped *v*-SiO_2_ indicate that Cl-doped *v*-SiO_2_ is consistently elastically stiffer than F-doped *v*-SiO_2_.
3. The difference in elastic moduli between F- and Cl-doped *v*-SiO_2_ may be due to buttressing of the rigid polyhedral network by the larger Cl atom. Unlike the elastic moduli of halogen-doped *v*-SiO_2_, that of OH-doped *v*-SiO_2_ is not consistently elastically softer or stiffer relative to the halogen- doped *v*-SiO_2_. The elastic moduli and wave speed reductions for OH-doped *v*-SiO_2_ have more compliant *K*_S_ and *V*_P_, but are more resistant to shear (lower *G*, *E*, and *V*_S_).
4. Most notably, OH-doped *v*-SiO_2_ has a decreasing Poisson’s ratio with increasing OH, which corresponds to a more open silicate network. Halogen-doped *v*-SiO_2_ exhibits the opposite trend in Poisson’s ratio, indicative of a more closely packed network.
5. Raman spectroscopy and MD simulations support the structural interpretation of the trends in Poisson’s ratio data for all doped *v*-SiO_2_ in this study. Halogen-doped *v*-SiO_2_ shows decreases in ≥5-member rings and increases in 3- and 4-member rings, whereas OH-doped *v*-SiO_2_ shows increases in ≥5-member rings and decreases in 3- and 4-member rings.
6. *v*-SiO_2_ accommodates network stresses by the bending of intertetrahedral angles. Increased populations of ≥5-member rings in OH-doped *v*-SiO_2_ can accommodate more stress than halogen-doped *v*-SiO_2_, which has increased populations of 3- and 4-member rings. This implies that halogen-doped *v*-SiO_2_ will deform less than OH-doped *v*-SiO_2_ under high stresses or pressures.
7. F is shown to increase isothermal compressibility the most at the concentrations measured in this study, which corresponds to a 10% increase in Rayleigh scattering up to 5 mol % F. Cl (up to 3.67 mol %) and OH (up to 0.42 mol %) follow at 1 and 1.4% increases in Rayleigh scattering, respectively, and are within error. As a direct consequence of the relative stiffness Cl imparts on *v*-SiO_2_, it effectively reduces the isothermal compressibility contribution to Rayleigh scattering and is more resistant to stress-induced deformation.

## Supplementary Material







## Notes

Tabulated data of fictive temperature, elastic properties, GHz measurements, and from literature; figures showing combined results across dopant species, Raman spectra inset, and energy versus distance curve for MD simulations; MD simulation summary of methods; FTIR data (2260 cm^–1^ peak) of F-doped and Cl-doped *v*-SiO_2_; Boson peak from Raman spectra of F- and Cl-doped *v*-SiO_2_; MD supplement including model parameters (PDF)
Fluorine composition by EPMA (XLSX)
Chlorine composition by EPMA (XLSX)
