# Supplementary material for: Chemical Species-Dependent Structural Modification of Vitreous SiO2 by Monovalent Anions
Source: J Phys Chem B. 2026 Mar 19;130(13):3740–53. doi: 10.1021/acs.jpcb.5c07097 (PMC13051433; doi:10.1021/acs.jpcb.5c07097)
Supplement: Supplementary file 1 [file jp5c07097_si_001.pdf]

Supporting Information for Publication

Chemical Species-Dependent Structural Modification of Vitreous SiO_2 by Monovalent Anions

AUTHOR NAMES. *Lindsay M. Harrison,*[a*] *Alisha N. Clark*[a*], *Adam R. Sarafian*[b], *Lisa A. Moore*[b], *Craig D. Nie*[b], *Galan G. Moore*[b], *James E. Tingley*[b], *Matthew E. McKenzie*[b]

*lindsay.harrison@colorado.edu

*alisha.clark@colorado.edu

[a]Department of Earth Science, University of Colorado, Boulder, Boulder, CO 80309, USA

[b]Corning Incorporated, Science & Technology, Corning, NY 14831, USA

Table of Contents

Tables S1-S4 .... S3-S7
Table S1: Fictive temperature S3
Table S2: Tabulated data S4
Table S3: Literature tabulated data S5
Table S4: Gigahertz ultrasonic interferometry S6

Figures S1-S8 .... S7-S14
Figure S1: Density S7
Figure S2: Young's modulus S8
Figure S3: Shear modulus S9
Figure S4: Bulk modulus S10
Figure S5: Poisson's ratio S11
Figure S6: FTIR data (2260 cm^{-1} peak) of F-doped *v*-SiO_2 .... S12
Figure S7: FTIR data (2260 cm^{-1} peak) of Cl-doped *v*-SiO_2 .... S13
Figure S8: Boson peak from Raman spectra of F- and Cl-doped *v*-SiO_2 .... S14

Molecular dynamics supplement S15
Table S5: Model parameters S15
Figure S9 .... S16

Table S1: Fictive temperature and density calculations

Dopant	wt %	mol %	Peak location (cm^{-1})	T_f (°C)	Calculated density (g/cm^3)	Actual density (g/cm^3)
Cl	0.12	0.20	2267.2	863	2.200	2.203
Cl	0.77	1.30	2268.3	831	2.200	2.2
Cl	1.32	2.22	2269.2	807	2.200	2.198
Cl	1.55	2.60	2269.4	802	2.199	2.196
Cl	2.2	3.67	2269.9	789	2.199	2.194
F	0.2	0.63	2262.1	1036	2.202	
F	0.3	0.94	2267.5	854	2.200	2.201
F	0.74	2.30	2268.3	831	2.200	2.197
F	0.91	2.82	2269	812	2.200	2.197
F	1.03	3.19	2268.2	834	2.200	2.195
F	1.1	3.40	2270	778	2.199	2.195
F	1.25	3.85	2272.4	728	2.199	2.191
F	1.9	5.77	2273.2	710	2.199	2.19

Table S2. Compositions, measured and calculated elastic moduli, acoustic wave speeds, and densities of *v*-SiO_2.

Dopant	Total dopant (wt%)	Total dopant (mol%)	Young's modulus (GPa)	Shear modulus (GPa)	Bulk modulus (GPa)	Poisson's ratio	V_P (m/s)	V_S (m/s)	Density (g/cm^3)	T_f (˚C)
F	0.2	0.63 (±0.04)	72.0 (±1.4)	30.9 (±0.6)	35.7 (±0.7)	0.164 (±0.007)	-	-	-	1036
F	0.3	0.94 (±0.12)	71.8 (±1.4)	30.8 (±0.6)	35.9 (±0.7)	0.166 (±0.007)	5890 (±5)	3730 (±2)	2.201	854
F	0.74	2.30 (±0.06)	69.7 (±1.4)	29.8 (±0.6)	35.0 (±0.7)	0.168 (±0.007)	-	-	2.197	831
F	0.91	2.82 (±0.05)	68.9 (±1.4)	29.6 (±0.6)	34.3 (±0.7)	0.165 (±0.007)	5810 (±2)	3636 (±1)	2.197	812
F	1.03	3.19 (±0.13)	68.1 (±1.4)	29.0 (±0.6)	34.6 (±0.7)	0.172 (±0.007)	-	-	2.195	834
F	1.1	3.40 (±0.14)	68.0 (±1.4)	29.1 (±0.6)	34.5 (±0.7)	0.171 (±0.007)	-	-	2.195	778
F	1.25	3.85 (±0.22)	67.0 (±1.3)	28.6 (±0.6)	34.2 (±0.7)	0.173 (±0.007)	5750 (±4)	3592 (±1)	2.191	728
F	1.9	5.77 (±0.34)	64.7 (±1.3)	27.6 (±0.6)	32.7 (±0.7)	0.17 (±0.007)	5659 (±4)	3768 (±2)	2.190	710
Cl	0.12	0.20 (±0.02)	72.9 (±1.5)	31.3 (±0.6)	36.3 (±0.7)	0.165 (±0.007)	5944 (±1)	3742 (±1)	2.203	863
Cl	0.77	1.21 (±0.24)	72.3 (±1.4)	31.0 (±0.6)	36.1 (±0.7)	0.166 (±0.007)	-	-	2.200	831
Cl	1.32	2.19 (±0.06)	71.1 (±1.4)	30.4 (±0.6)	35.7 (±0.7)	0.168 (±0.007)	5915 (±6)	3706 (±1)	2.198	807
Cl	1.55	2.58 (±0.05)	70.5 (±1.4)	30.1 (±0.6)	35.5 (±0.7)	0.169 (±0.007)	-	-	2.196	802
Cl	2.2	3.67 (±0.07)	69.7 (±1.4)	29.7 (±0.6)	35.7 (±0.7)	0.174 (±0.007)	5834 (±10)	3645 (±6)	2.194	789
OH	0.0009	0.00	73.1 (±1.5)	31.5 (±0.6)	35.8 (±0.7)	0.16 (±0.006)	5955 (±0.1)	3770 (±1)	2.190	-
OH	0.014	0.05	73.0 (±1.5)	31.5 (±0.6)	35.6 (±0.7)	0.158 (±0.006)	5945 (±0.2)	3761 (±0.4)	2.201	-
OH	0.031	0.11	72.9 (±1.5)	31.4 (±0.6)	35.6 (±0.7)	0.159 (±0.006)	5950 (±0.5)	3762 (±1)	2.201	-
OH	0.052	0.18	73.0 (±1.5)	31.5 (±0.6)	35.7 (±0.7)	0.159 (±0.006)	-	3771 (±1)	2.201	-
OH	0.084	0.30	72.8 (±1.5)	31.4 (±0.6)	35.5 (±0.7)	0.158 (±0.006)	5956 (±2)	3754 (±0.5)	2.200	-
OH	0.1	0.35	73.0 (±1.5)	31.6 (±0.6)	35.4 (±0.7)	0.156 (±0.006)	5926 (±1)	3754 (±1)	2.201	976
OH	0.12	0.42	72.9 (±1.5)	31.5 (±0.6)	35.5 (±0.7)	0.158 (±0.006)	5919 (±2)	3756 (±1)	2.200	-
Undoped	0	0.00	73.3 (±1.5)	31.6 (±0.6)	36.0 (±0.7)	0.161 (±0.006)	5943 (±0.1)	3767 (±1)	2.200	890
Undoped (MD)	0	0.00	72.0	30.4	37.9	0.184	-	-	-	-

Table S3. Tabulated data from the literature to compare to our own.

Dopant	Total dopant (mol%)	Young's modulus (GPa)	Shear modulus (GPa)	Bulk modulus (GPa)	Poisson's ratio	V_P (m/s)	V_S (m/s)	Density (g/cm^3)	Reference
F	0	74.58	31.7	38.39	0.176	6049	3792	2.204	Hirao et al., 1991
F	1.81	72.91	31.19	36.39	0.169	5959	3766	2.199	
F	2.67	72.53	31.1	36.20	0.166	5945	3762	2.197	
F	9.46	59.25	24.94	31.62	0.187	5457	3384	2.179	
Cl	0	72.96	31.28	36.43	0.166	5957	3769	2.195	Kushibiki et al., 2000
Cl	0.25	72.76	31.09	36.75	0.170	5959	3757	2.195	
OH	1.05	70.83	30.42	35.21	0.164	5866	3734	2.200	LeParc et al., 2006

Table S4: Gigahertz ultrasonic interferometry sample attributes and measurements

Dopant	mol %	Thickness (mm)	P-wave traveltime (s)	V_P (m/s)	S-wave traveltime (s)	V_S (m/s)
F	0.94	0.960	3.26E-07	5890 (±5)	5.16E-07	3720 (±2)
F	2.8	0.587	2.02E-07	5810 (±2)	5.97E-07	3636 (±1)
F	3.85	0.648	2.25E-07	5750 (±4)	3.61E-07	3592 (±1)
F	5.77	0.648	2.29E-07	5659 (±4)	3.23E-07	3768 (±2)
Cl	0.20	0.904	3.04E-07	5944 (±1)	4.83E-07	3742 (±1)
Cl	2.22	1.017	3.44E-07	5915 (±6)	5.49E-07	3706 (±1)
Cl	3.67	0.843	2.98E-07	5834 (±10)	4.62E-07	3645 (±6)
OH	0.00	1.038	3.49E-07	5955 (±0.1)	5.51E-07	3770 (±1)
OH	0.05	0.911	3.06E-07	5945 (±0.2)	4.83E-07	3761 (±0.4)
OH	0.11	0.921	3.09E-07	5950 (±0.5)	4.89E-07	3762 (±1)
OH	0.18	0.938	-	-	4.98E-07	3771 (±1)
OH	0.30	0.990	3.32E-07	5956 (±2)	5.25E-07	3754 (±0.5)
OH	0.35	0.991	3.35E-07	5926 (±1)	5.28E-07	3754 (±1)
OH	0.42	1.001	3.38E-07	5919 (±2)	5.33E-07	3756 (±1)
Undoped	0	0.989	3.33E-07	5943 (±0.1)	5.25E-07	3767 (±1)

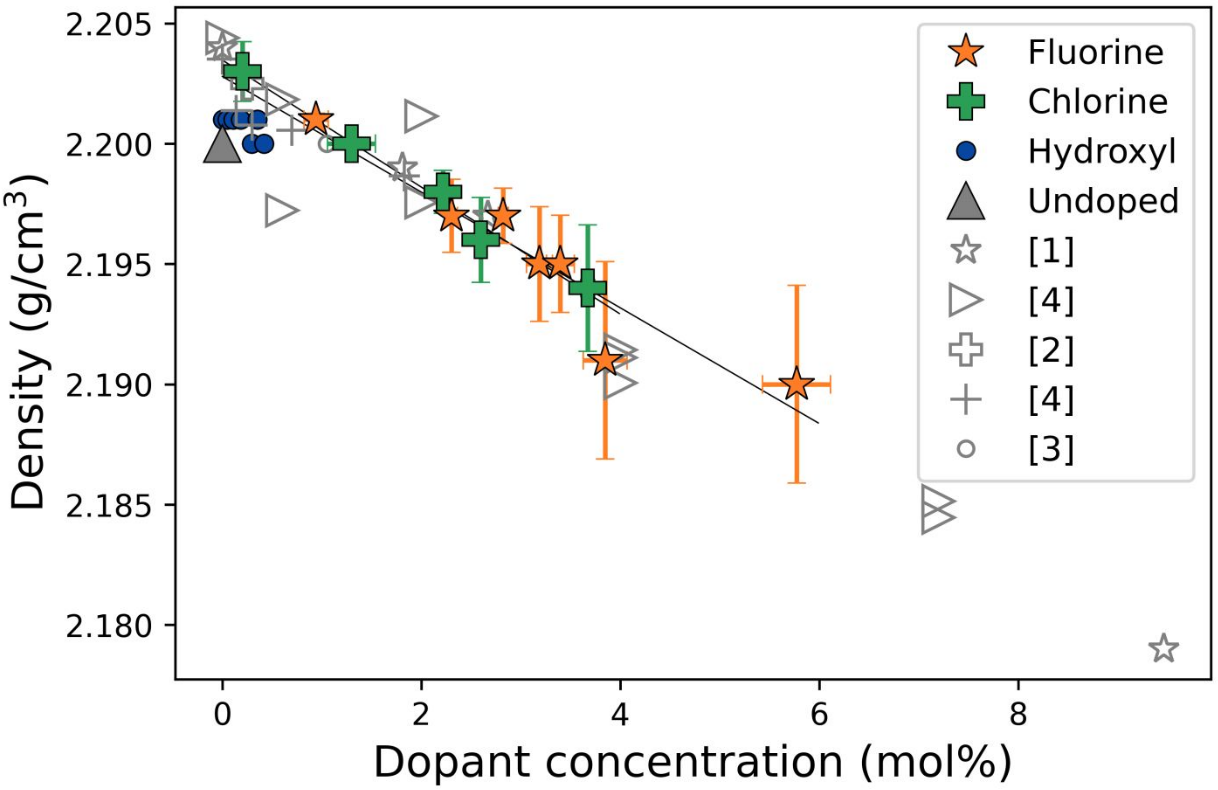

Figure S1. Density measured by Archimedes method as a function of mol% dopant of F (orange stars), Cl (green crosses), and OH (blue circles). Density determined by Archimedes method for F, Cl, and OH [1–3] and the heavy solution method for F and Cl[4] are also shown. The ultrapure endmember, HPFS 8655, is plotted in a gray triangle for comparison to all doped silicas.

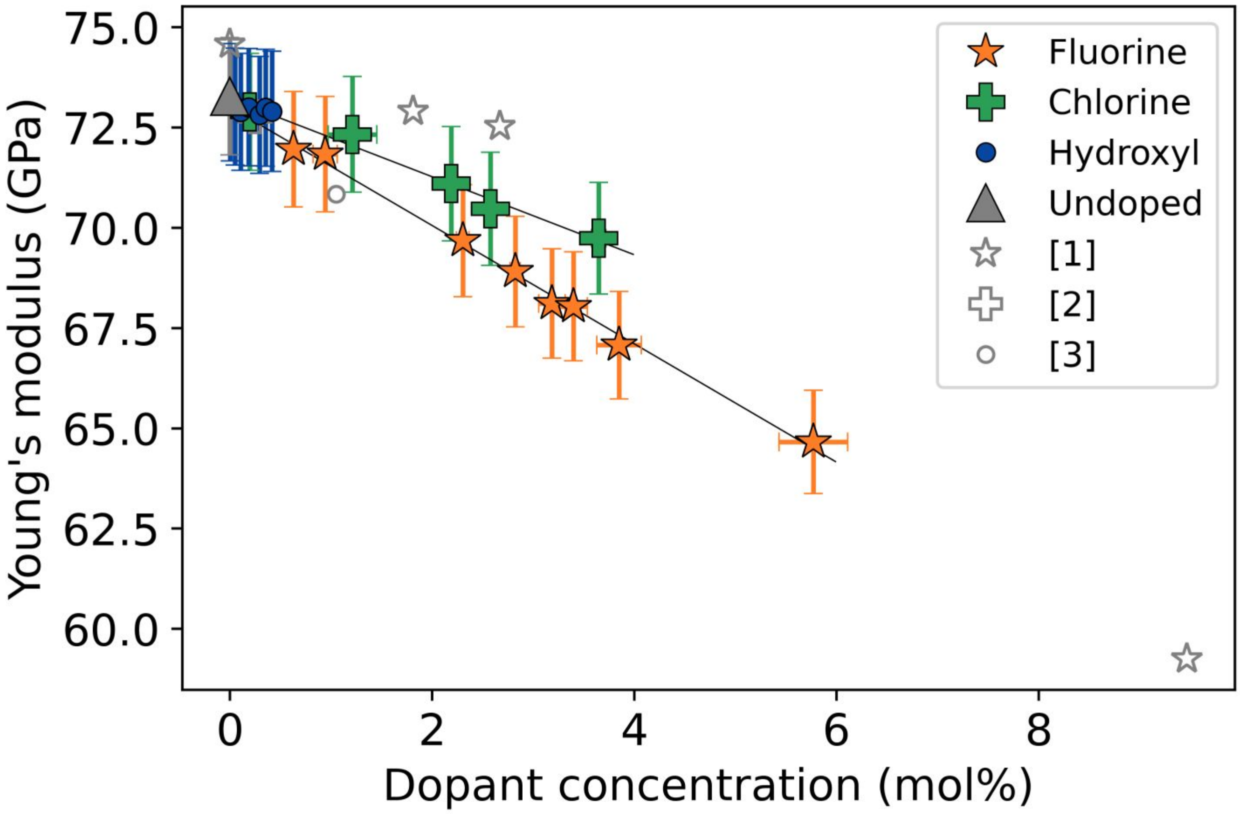

Figure S2. Young's modulus measured by RUS for v-SiO_2 as a function of mol% dopant at one-atmosphere and room temperature. F (orange stars), Cl (green crosses), and OH (blue circles). Literature values for Young's modulus for v-SiO_2 doped with F (ultrasonic resonance[1]), Cl (ultrasonic microspectroscopy[2]), and OH (Brillouin scattering[3]), are also shown. Young's and shear moduli are obtained from the elastic tensor determined by RUS.

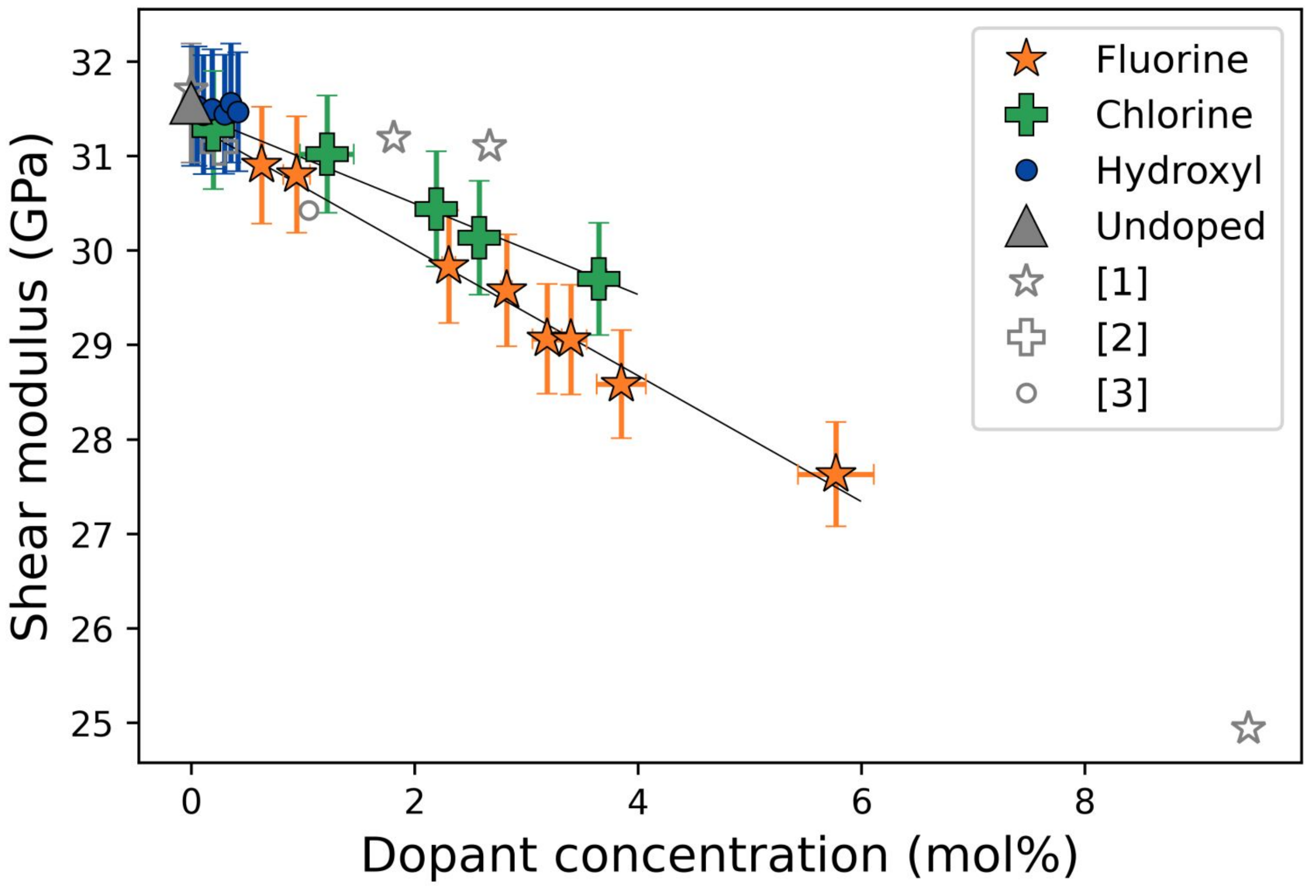

Figure S3. Shear modulus measured by RUS for v-SiO_2 as a function of mol% dopant at one-atmosphere and room temperature. F (orange stars), Cl (green crosses), and OH (blue circles). Literature values for shear modulus for v-SiO_2 doped with F (ultrasonic resonance[1]), Cl (ultrasonic microspectroscopy[2]), and OH (Brillouin scattering[3]), are also shown.

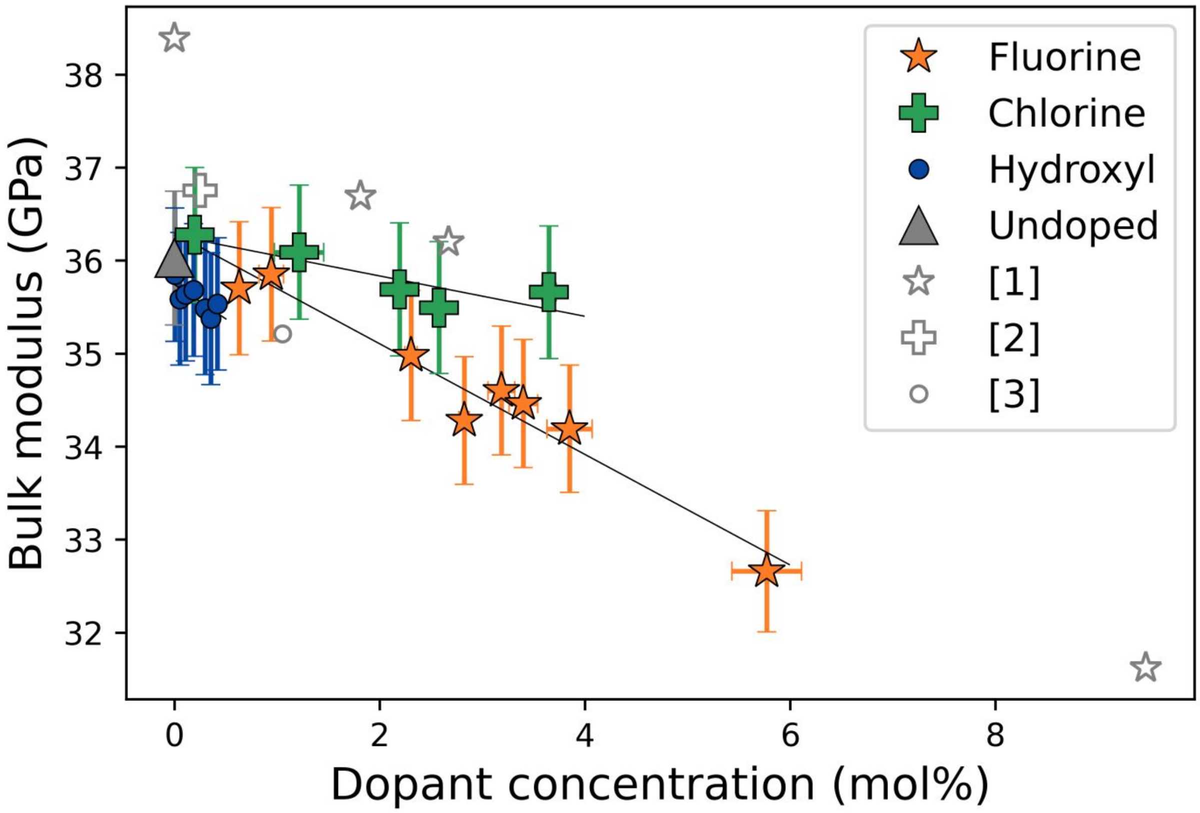

Figure S4. Bulk modulus as a function of dopant determined by RUS. F (orange stars), Cl (green crosses), and OH (blue circles). Literature values for bulk modulus for *v*-SiO_2 doped with F (ultrasonic resonance[1]), Cl (ultrasonic microspectroscopy[2]), and OH (Brillouin scattering[3]), are also shown.

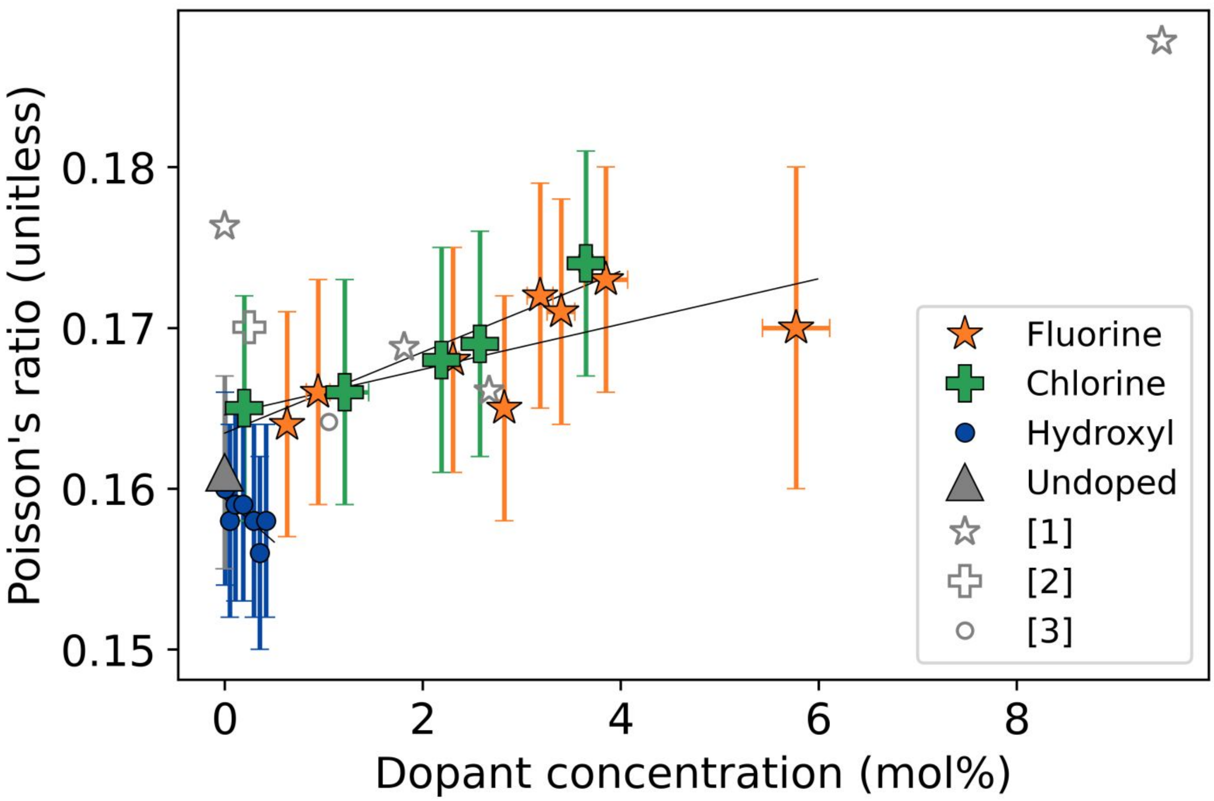

Figure S5. Poisson's ratio for v-SiO_2 doped with F (orange stars), Cl (green crosses), and OH (blue circles). Literature values of Poisson's for v-SiO_2 doped with F (ultrasonic resonance[1]), Cl (ultrasonic microspectroscopy[2]), and OH (Brillouin scattering[3]), are also shown. Poisson's ratio is calculated from the reported values of Young's and shear moduli.

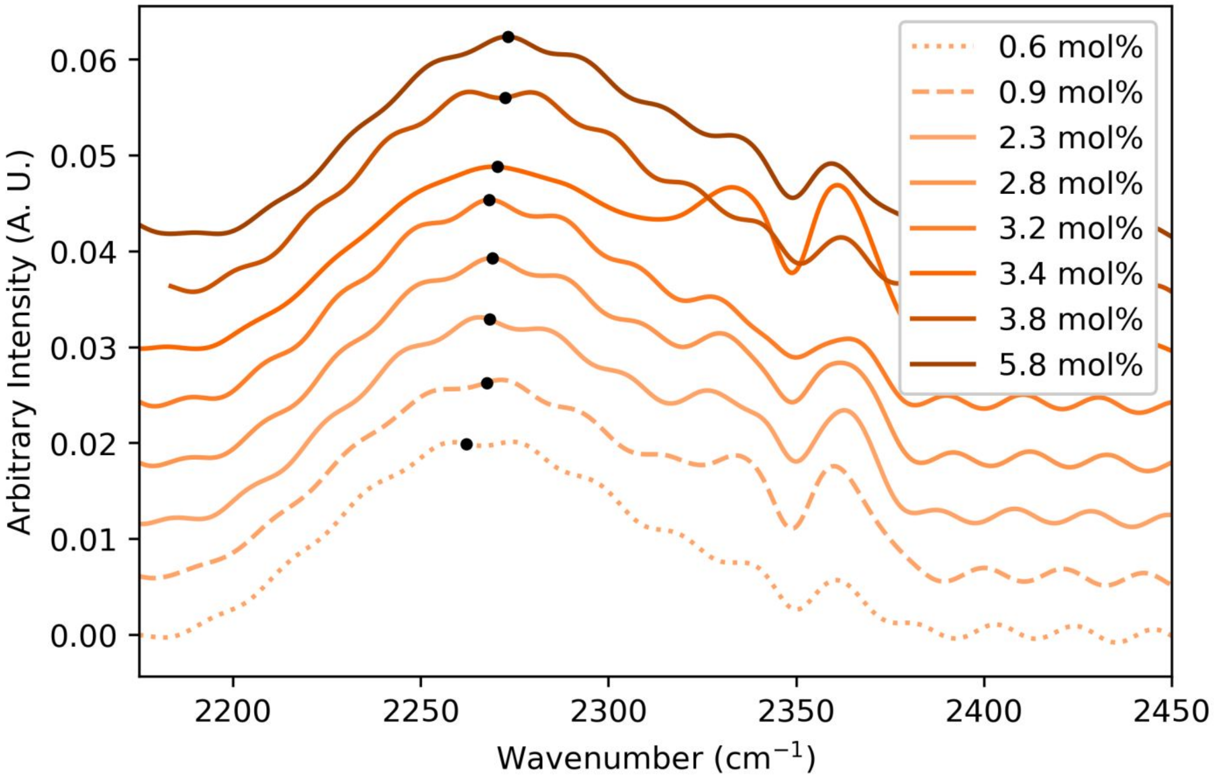

Figure S6. FTIR of the 2260 cm^{-1} peak location for F-doped v-SiO_2 with arbitrary intensity offset to emphasize shift of peak with increasing F concentration. Peak locations used to determine T_f are labeled and are shown with their corresponding samples in Table ST1. This data contains much more noise compared to the Cl-doped v-SiO2 samples as the F samples were thinner (~0.1 mm), and signal is proportional to thickness as shown by the relationship $d * \rho \propto absorbance$ (Supplemental Material eq. 1) based on equation 1 in [5] where absorbance is the height of the absorption peak, d is the thickness of the specimen, and ρ is density.

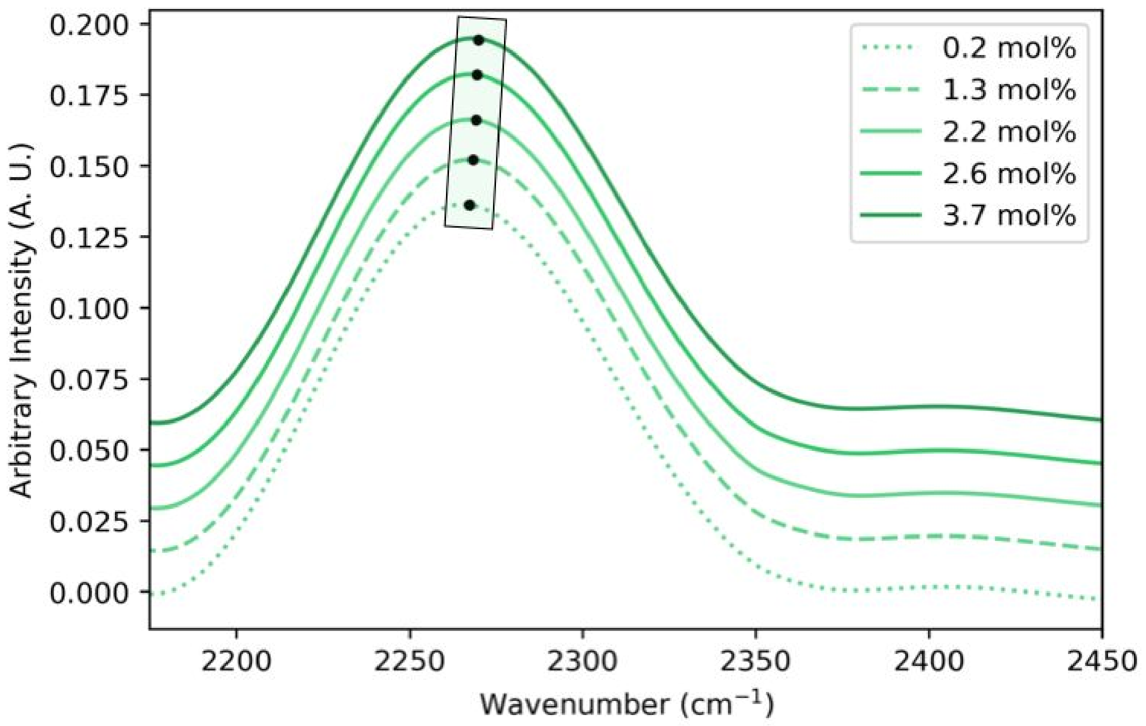

Figure S7. FTIR of the 2260 cm^{-1} peak location for Cl-doped *v*-SiO_2. Peak locations used to determine T_f are labeled and are shown with their corresponding samples in Table ST1. As mentioned in the caption of Figure S8, these samples were thicker than the F-doped samples, with polishing to ~1mm, showing much less noise.

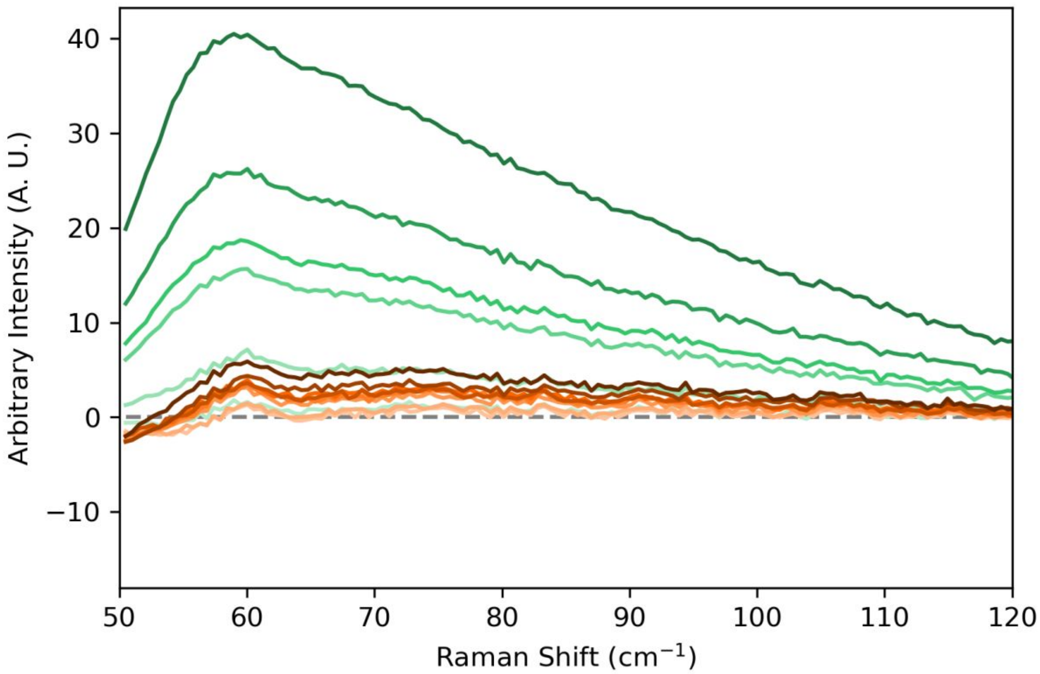

Figure S8. Boson peak comparison. Orange lines represent F- doped *v*-SiO_2 and green lines represent Cl-doped *v*-SiO_2. Color intensity corresponds to relative concentration of the sample.

Model supplement

To accurately model the interactions of dopants (F, Cl, and OH) in the *v*-SiO_2 glass system, modifications to the PMMCS force field were required. To make the force field somewhat commensurate with these elements, we decided to use a charged Lennard-Jones potential. Lennard-Jones (LJ) parameters were derived for fluorine, chlorine, and hydrogen using quantum mechanical calculations performed in Gaussian 16 with the B3LYP functional and AUG-cc-pVTZ basis set. Energy vs. separation curves were calculated for model clusters of $(OH)_3$-Si-X, where X represents F, Cl, or OH, with the $(OH)_3$ group mimicking the three bridging oxygens typical of the silica glass network. The LJ parameters were fit to these curves, with sigma (σ) adjusted to match experimental atomic separations and epsilon (ε, the potential well depth) calibrated to ensure compatibility with quantum mechanical results. These parameters were then tested and verified in LAMMPS simulations. While the modifications were completed in under a month for expediency, further refinement would require additional experimental and computational data, particularly due to the low concentrations of dopants in the system, which complicates force field development and validation. The final LJ parameters for F, Cl, and H are provided in Table below, and the energy vs. distance curves illustrating the fitted potentials are shown in Figure S9 below.

Table S5: Element pair and LJ parameters.

Element Pair	Lennard Jones ε (eV)	Lennard Jones σ (Å)
$Si^{+2.4}$ – $Cl^{-0.6}$	3.949	2.08
$Si^{+2.4}$ – $F^{-0.6}$	5.973	1.7
$O^{-1.2}$ – $H^{+0.6}$	4.790	0.96

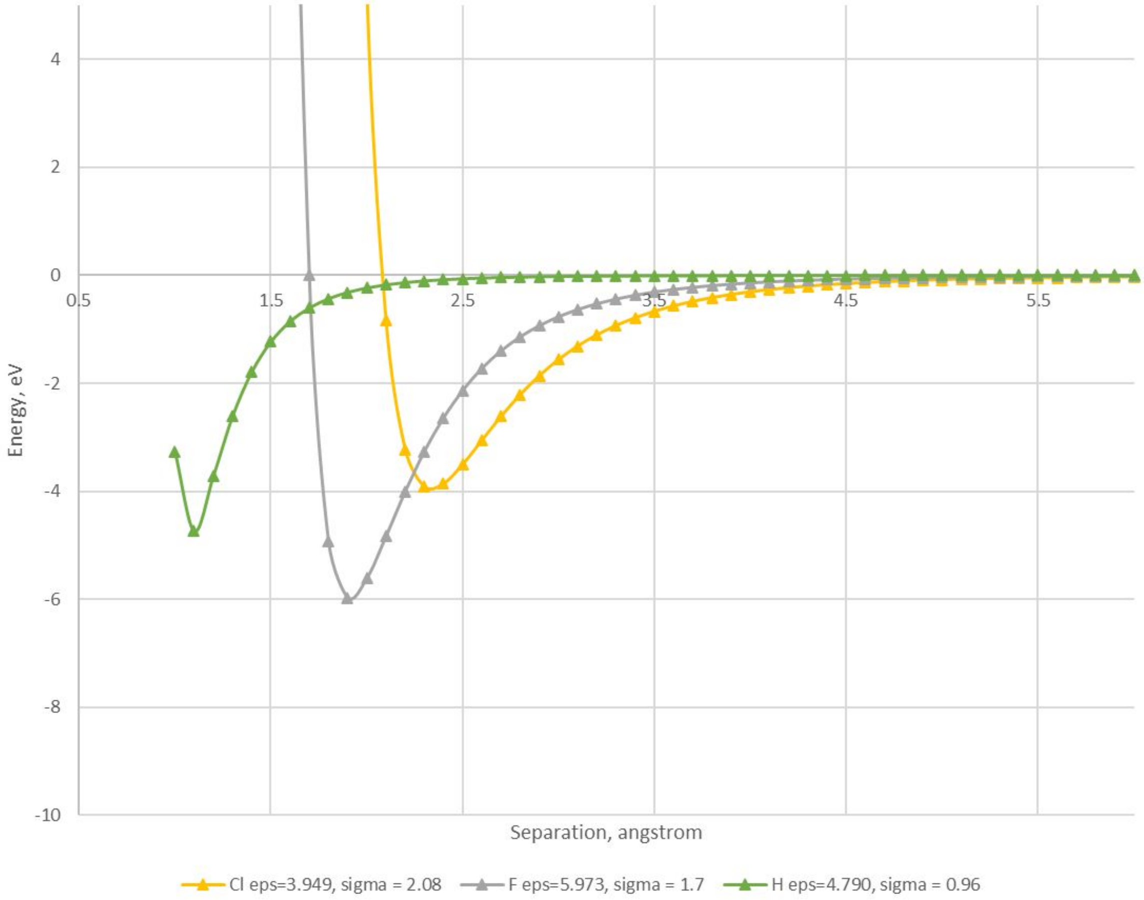

Figure S9. Energy versus distance curve for the F, Cl, and H model clusters showing fitted potentials.

References

(1) Hirao K.; Kawano T.; Tanaka K.; Soga N. Elastic Anomaly and Structure of F-Doped Silica Glass. *J. Ceram. Soc. Japan* **1991**, *99* (1151), 600–607. https://doi.org/10.2109/jcersj.99.600.

(2) Kushibiki, J.; Wei, T.-C.; Ohashi, Y.; Tada, A. Ultrasonic Microspectroscopy Characterization of Silica Glass. *Journal of Applied Physics* **2000**, *87* (6), 3113–3121. https://doi.org/10.1063/1.372307.

(3) Le Parc, R.; Levelut, C.; Pelous, J.; Martinez, V.; Champagnon, B. Influence of Fictive Temperature and Composition of Silica Glass on Anomalous Elastic Behaviour. *J. Phys.: Condens. Matter* **2006**, *18* (32), 7507. https://doi.org/10.1088/0953-8984/18/32/001.

(4) Kakiuchida, H.; Shimodaira, N.; Sekiya, E. H.; Saito, K.; Ikushima, A. J. Refractive Index and Density in F- and Cl-Doped Silica Glasses. *Applied Physics Letters* **2005**, *86* (16), 161907. https://doi.org/10.1063/1.1897062.

(5) Stolper, E. Water in Silicate Glasses: An Infrared Spectroscopic Study. *Contr. Mineral. and Petrol.* **1982**, *81* (1), 1–17. https://doi.org/10.1007/BF00371154.
